# Increased expression of the PIEZO2 mechanoreceptor in fibroblasts and endothelial cells within the lymphatic and vascular vessels of keloids

**DOI:** 10.1002/path.6455

**Published:** 2025-07-31

**Authors:** Shinsuke Akita, Sanae Ikehara, Masahiro Kiuchi, Kota Kokubo, Kazuhiko Azuma, Syouta Ohki, Hiroyuki Matsuyama, Joceline Theda Kadarman, Yohei Hosokawa, Yoshihiro Akimoto, Yosuke Inaba, Hideki Hanaoka, Nobuyuki Mitsukawa, Kiyoshi Hirahara, Toshinori Nakayama, Yuzuru Ikehara

**Affiliations:** ^1^ Department of Plastic, Reconstructive, and Aesthetic Surgery, Graduate School of Medicine Chiba University Chiba Japan; ^2^ Department of Pathology, Graduate School of Medicine Chiba University Chiba Japan; ^3^ Department of Immunology, Graduate School of Medicine Chiba University Chiba Japan; ^4^ Division of Pathology Kaihin Hospital Chiba Japan; ^5^ Department of Microscopic Anatomy Kyorin University School of Medicine Tokyo Japan; ^6^ Clinical Research Center Chiba University Hospital Chiba Japan; ^7^ AMED‐CREST, AMED Tokyo Japan

**Keywords:** keloids, mechanoreceptor, PIEZO2, fibroblasts, endothelial cells in lymphatic and vascular vessels

## Abstract

Keloids are scars that grow abnormally due to excessive extracellular matrix production by fibroblasts and increased angiogenesis. Chronic tension is implicated in their growth, but the exact pathology remains unclear. This study investigated the increased expression of molecules responsible for sensing pressure in keloids compared with lymphedema, which is also a non‐tumorous fibroproliferative disease caused by another etiology. Higher expression levels of *COL1A2*, *PIEZO2*, and *POSTN* were observed in the keloid group compared with the lymphedema group. *PIEZO2* expression levels showed a strong correlation with both *COL1A2* (*r* = 0.9252, 95% CI 0.8474–0.9641, *p* < 0.001) and *POSTN* (*r* = 0.9118, 95% CI 0.8213–0.9575, *p <* 0.001). Additionally, *PIEZO2* expression levels were significantly higher in recurrent keloids than in non‐recurrent keloids (3,032.5 ± 1,090.2 versus 1,241.9 ± 860.7, *p* = 0.032). Analysis of gene expression at the single‐cell level found upregulation of *PIEZO2* in vascular and lymphatic endothelial cells, and a subgroup of fibroblasts. Additionally, *COL1A1*, *COL1A2*, *COL3A1*, and *POSTN* expression was also increased in the fibroblast subgroup. Furthermore, in fibroblasts with high *PIEZO2* expression, extracellular matrix collagen production signaling was augmented. Histological analysis confirmed the presence of PIEZO2‐positive cells in the perivascular stroma active area of keloid tissue, together with inflammatory cells. Therefore, since PIEZO2‐positive cells are highly expressed specifically in keloids and are deeply involved in their recurrence and activity, we propose that the pathogenesis of keloids is constructed by PIEZO2‐positive cells. © 2025 The Author(s). *The Journal of Pathology* published by John Wiley & Sons Ltd on behalf of The Pathological Society of Great Britain and Ireland.

## Introduction

Keloids represent a pathological form of scar tissue, characterized by uncontrolled growth, although the underlying mechanisms involved in their persistent growth remain unclear. The unusual scar tissue in keloids is characterized by an increased number of fibroblasts and blood vessels, along with excess extracellular matrix production within the dermis [1]. The persistent growth of keloids has been associated with genetic factors, as previous studies have reported differences in the prevalence of keloids between ethnic groups and populations, and specific single nucleotide polymorphisms [2]. However, the ability to identify and predict the onset of keloids in patients using genomic information alone remains difficult. Although keloids can be differentiated from normal scar tissue and hypertrophic scars by their gross appearance, histological architecture, and gene expression profile in fibroblasts, the ability to diagnose keloids based on the presence of specific cells or biomarker molecules is challenging [1, 3].

Despite their non‐neoplastic nature, surgical approaches for the treatment of keloids are still under development. First‐line therapy typically involves corticosteroids, due to the inflammatory responses associated with keloids [4, 5]. In cases of uncontrolled growth, surgical resection may be required to manage the continuous growth of pathological scar tissue. Multimodal treatment approaches, combining surgical therapy and adjuvant therapy, including surgical resection, radiation, and 5‐fluorouracil (5‐FU), are commonly applied [4, 5, 6]. However, the recurrence rate of keloids, even after surgical therapy, combined with adjuvant therapy, remains between 10% and 30% [7].

Plastic surgery may be used to treat non‐tumorous fibroproliferative diseases of lymphedema, as well as keloids. Keloids show nodular fibroproliferation in areas chronically exposed to mechanical tension, and surgical treatment involves nodular excision. Conversely, lymphedema presents with diffuse fibroproliferation, resulting from congestion of the leaked lymph fluid, and its surgical treatment involves reconstruction of the lymphatic circulation [8]. Notably, an assumed factor promotes fibroproliferation in keloids in response to chronic mechanical tension, the promotive mechanism of which may differ from that of lymphedema. However, while earlier reports suggest a potential mechanism by which tension promotes proliferation in the fibrous tissue of keloids [9], little information is available regarding which cells sense mechanical pressure and which molecules are expressed in these cells [10].

This study investigated the increased expression of pressure sensing molecules in keloid fibrosis compared with lymphedema. We observed that higher expression levels of *PIEZO2* were correlated with keloid recurrence. The piezo‐type mechanosensitive ion channel family, composed of *PIEZO1* and *PIEZO2*, has been identified as a key mediator of cellular responses to mechanical pressure [11]. *PIEZO1* is present in different types of cells in both parenchyma and stroma, including cells found in hypertrophic scar tissue [12, 13, 14, 15]. In contrast, *PIEZO2* appears to have more restricted expression, limited to the neuronal lineage, such as sensory trigeminal ganglia, dorsal root ganglia, and neuroendocrine differentiated cells in the skin, bladder, and lung [16]. Therefore, to clarify which cells in keloids express *PIEZO2*, we analyzed gene expression at the single‐cell level and found that *PIEZO2* was upregulated in vascular endothelial cells (VECs), lymphatic endothelial cells (LECs), and a subgroup of fibroblasts that the extracellular matrix collagen production signaling augmented.

## Materials and methods

### Ethics approval and patient consent

All patients signed informed consent forms, and the study was approved by the Ethics Committee of the Chiba University Graduate School of Medicine and each participating hospital [registration numbers: 4235 (keloid analysis) and M10291 (fibrous diseases analysis)]. Written consent was obtained from all subjects to collect samples and information including clinical photographs.

### Human samples

Resection specimens from 26 patients who underwent surgical keloid treatment were analyzed at our institution between June 2018 and March 2023 (see supplementary material, Tables S1 and S2 for patient information). Both severe lymphedema (SL) and keloids exhibit fibroproliferation; however, mechanical tension solely contributes to the fibrotic development of keloids. In this study, we used SL as a control for non‐tension‐induced fibrosis to investigate the molecular mechanisms by which mechanical tension contributes to fibroproliferation in keloids. Additionally, mild lymphoedema (ML) cases, with minimal or no fibrosis, were included for comparison of the degree of advanced fibrosis in SL cases compared with ML cases. For single‐cell RNA sequencing (scRNA‐seq) analysis, we used tissue from standard unaffected skin, as excess skin from a local skin flap, typically used to cover the defect, is generally obtained from a site unaffected by surrounding mechanical tension.

### Diagnosis, clinical classification, surgical treatment indications, and recurrence assessment of keloids

Diagnosis and treatment planning for patients with keloids were conducted according to the Clinical Practice Guidelines set forth by the Japanese Society of Plastic and Reconstructive Surgery, using the Japan Scar Scale (JSS), a classification system for grading and selecting appropriate treatment methods for keloids and hypertrophic scars [17, 18]. The scores incorporate risk factors and current symptoms to classify patients into three risk categories based on a total score ranging from 0 to 25: low‐risk (0–5), intermediate‐risk (6–15), and high‐risk (16–25) [19]. Notably, keloid recurrence was defined as the presence of subjective symptoms and objective nodule regrowth observed over 2 years after keloid resection, requiring further treatment, such as steroid injections or surgery.

### Histological analyses and immunohistochemistry detection

Histological analysis was performed on diagnostic samples fixed in 10% neutral buffered formalin and embedded in paraffin. Serial sections (3 μm thick) were stained with hematoxylin and eosin (H&E) (Muto Pure Chemicals Co., Ltd., Tokyo, Japan). Immunohistochemical analysis was performed using Target Retrieval Solution High pH (Agilent, Santa Clara, CA, USA) and the following primary antibodies: rabbit anti‐PIEZO2 antibody (1 μg/ml; Proteintech, Rosemont, IL, USA), mouse anti‐podoplanin monoclonal antibody (1:100 dilution; D2‐40; Signet Testing Labs Inc., Hayward, CA, USA), and rabbit anti‐periostin polyclonal antibody (1 μg/ml; Proteintech). Specific detection of primary antibodies was visualized using eFluor™660‐labeled mouse anti‐smooth muscle actin monoclonal antibody (1 μg/ml; Thermo Fisher Scientific, Waltham, MA, USA) as described previously [20, 21]. For detection of multiple primary antibodies, the Tyramide SuperBoost™ Kit for goat anti‐mouse IgG and anti‐rabbit IgG antibodies (Thermo Fisher Scientific), including Alexa Fluor™ 488, Alexa Fluor™ 555, and Alexa Fluor™ 647‐labeled solutions (Thermo Fisher Scientific), was applied. Fluorescent immunostaining was observed using confocal microscopy (LSM 800; Carl Zeiss, Oberkochen, Germany), while whole slide images of tissue sections with H&E staining were obtained using a NanoZoomer S20 (Hamamatsu Photonics K.K., Hamamatsu, Japan).

### Quantification of RNA expression and scRNA‐seq analyses

Details of RNA sequencing and analysis data from fibrous disease patients are provided in Supplementary materials and methods. Single‐cell suspensions were prepared from resected keloid tissue and scRNA‐seq analysis was performed using Seurat [22]. We applied the R package CellChat (version 1.6.1, https://github.com/sqjin/CellChat, accessed 16 April 2025) [23] to analyze cell–cell communication networks within the scRNA‐seq data.

### Ultrastructural analysis using transmission electron microscopy (TEM)

Samples were fixed in phosphate‐buffered 2.5% glutaraldehyde (FUJIFILM Wako, Osaka, Japan) for 24 h and post‐fixed in 1% osmium tetroxide (FUJIFILM Wako) for 1 h, followed by 2% osmium tetroxide in 0.1 m phosphate buffer (pH 7.4) for 3 h, in an ice bath. Epoxy resin‐embedded samples were sectioned into 50–90 nm ultrathin sections, placed on gold mesh grids (Nisshin EM, Tokyo, Japan), and incubated with uranyl acetate (Merck, Darmstadt, Germany) for 10 min, followed by lead staining solution (Electron Microscopy Sciences, Hartfield, PA, USA) for 5 min [24]. Transmission electron microscopy (TEM) (JEM‐1200 EX; JEOL, Tokyo, Japan) was used to examine the ultrathin sections on gold mesh grids as described previously [24, 25].

### Statistical analyses

Statistical analyses were performed using JMP version 13 software (SAS Institute, Cary, NC, USA). We employed the Kruskal–Wallis and Wilcoxon tests to compare relative RNA expression levels among the ML, SL, and keloid groups. Pearson's correlation coefficients and 95% confidence intervals for all 30 cases were calculated and visualized using scatter plots of continuous variables. Keloid cases were divided into two groups: (1) four recurrent cases and six non‐recurrent cases, and (2) five cases with high expression of *PIEZO2* and five other cases. Post‐treatment outcomes were then compared between the groups. Continuous variables were analyzed using an unpaired Student's *t*‐test. Fisher's exact test was used to compare the frequency rate after treatment between groups when five or fewer items were present. A *p* value of < 0.05 was considered statistically significant.

## Results

### *PIEZO2* expression is augmented in keloid cases compared with lymphedema cases

**Figure 1:**
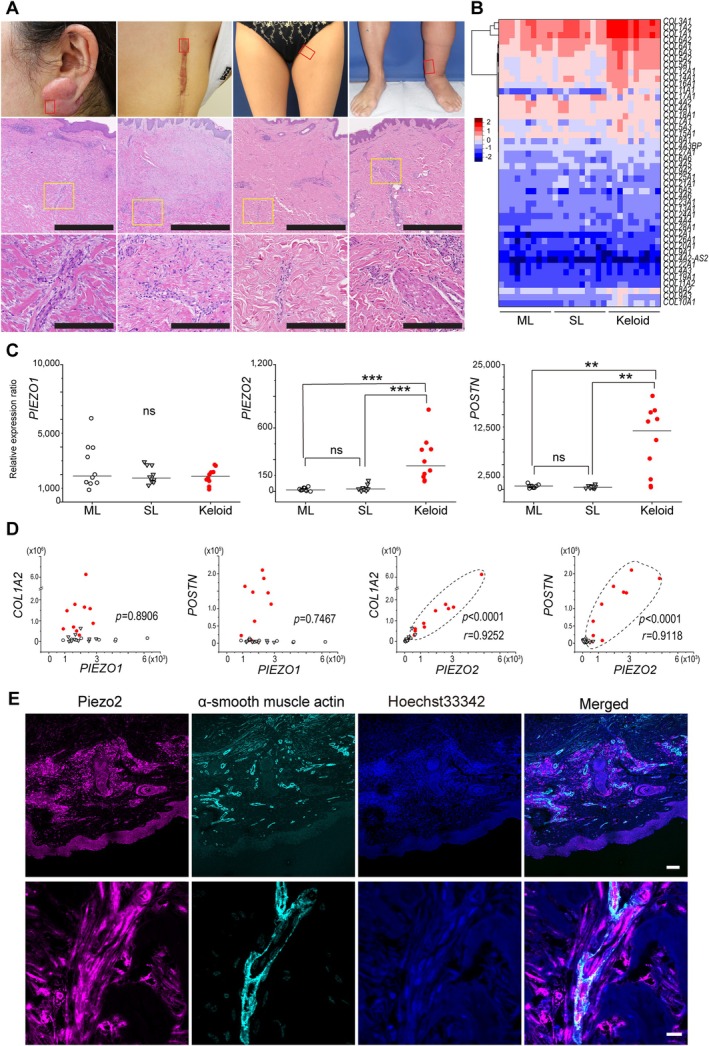
Enhanced expression of *PIEZO2* in keloid patients. (A–E) Relative expression levels of *PIEZO* and keloid‐related genes between the mild lymphedema (ML), severe lymphedema (SL), and keloid groups. (A) Written consent was obtained from all patients including for use of photographs. Representative H&E‐stained images are shown. The degree of fibrosis consistent with each disease was observed. Immune cells were observed in all three groups. Cases, from left to right, are KL1, KL4, ML8, and SL5 (details are provided in supplementary material, Table S1). Scale bars, 1,000 and 250 μm for black lines in the middle and lower panels, respectively. (B) *COL3A1*, *COL1A2*, and *COL1A1* were commonly highly expressed in all groups, with expression levels in the keloid group higher than those in the other two groups. (C) Relative expression ratios of *PIEZO1*, *PIEZO2*, and *POSTN* among the three groups. *PIEZO1* expression was not significantly different (*p* = 0.83 by Kruskal–Wallis test). *PIEZO2* and *POSTN* showed higher expression levels in the keloid group than in the other two groups (Wilcoxon test). ns, not significant. ***p < 0.001, **p < 0.01. White circles: ML; white triangles: SL; red circles: keloid. The Trimmed Mean of *M*‐values (TMM) normalization process was used for normalizing RNA‐seq count data. (D) Scatter plots show the correlation between *PIEZO1* and *PIEZO2* expression levels and *COL1A2* and *POSTN* expression levels. No significant correlation was observed between *PIEZO1* expression and either *COL1A2* or *POSTN. PIEZO2* expression levels showed strong correlation coefficients with *COL1A2* (*R =* 0.9252, 95% CI 0.8474–0.9641, *p* < 0.001) and *POSTN* (*R* = 0.9118, 95% CI 0.8213–0.9575, *p* < 0.001), respectively. White circles: ML; white triangles: SL; red circles: keloid. The keloid specimen is surrounded by a dashed line. (E) Immunostaining findings of keloid proliferative sites: PIEZO2‐positive cells are commonly found in SMA‐positive vascular walls and perivascular interstitial cells. Scale bars, 100 μm and 10 μm for the upper and lower panels, respectively.

To determine whether the expression of mechanosensitive channels is increased in keloid tissue compared with skin tissues from lymphedemas, we investigated ten keloid cases, ten ML cases, and ten SL cases (supplementary material, Table S1, cases KL1–KL10, SL1–SL10, and ML1–ML10). The selection criteria for keloid cases included a clinical history of persistent proliferative phase of the scar for more than 12 months under conservative treatment. In contrast, the selection criteria for lymphedema cases included a persistent tendency to peripheral enlargement and proliferation of fibrous tissues without scaring reactions. The control group included ten SL cases classified as stage II or later, and ten ML cases with clinically normal lymphatic function. Each keloid, ML, and SL exhibited grossly visible fibrotic appearances and fibrous‐rich tissue on histological examination (Figure 1A). Furthermore, infiltration of inflammatory cells was present to varying extents in resected tissues of all cases in the three groups, suggesting the involvement of inflammation in the development of fibro‐collagenous proliferation.

Next, we performed a global evaluation of the gene expression in resected skin tissue from these 30 cases. We clustered collagen gene expression in keloids and lymphedema and found that keloid tissue tended to have higher expression of *COL3A1*, *COL1A2*, *COL1A1*, *COL6A1*, and *COL5A1* than did the ML and SL groups (Figure 1B). However, no collagen genes were identified that were expressed only in the keloid group or only in the lymphedema groups.

Moreover, to investigate changes in the expression of the two mechanosensitive channels, we compared the *POSTN* expression previously reported to increase keloids [1] with *PIEZO1* and *PIEZO2* expression in the same 30‐case dataset. *PIEZO2* expression levels were significantly higher in the keloid group than in the ML and SL groups. In addition, the high *POSTN* expression in the keloid group was similar to findings in the previous study (Figure 1C and supplementary material, Tables S3–S5) [1]. In the ML group, several cases of high *PIEZO1* expression were observed. However, there was no significant difference in the *PIEZO1* expression levels between the three groups, and no significant correlation between *PIEZO1* expression levels and either *COL1A2* or *POSTN* (supplementary material, Table S6 and Figure 1D). In contrast, *PIEZO2* expression showed a strong positive correlation with the upregulation of *COL1A2* and *POSTN* (supplementary material, Table S6 and Figure 1D). These results show increased *PIEZO2* expression in keloid cases compared with lymphedema cases, and this trend was analogous to *COL1A2* and *POSTN* expression in keloid tissue. To validate this finding, *PIEZO2* expression in keloid tissue was confirmed by multiple fluorescence staining, which showed that the PIEZO2 antibody signal was distributed in both vascular and fibrous tissue in the dermis (Figure 1E and supplementary material, Figure S1).

### Clinical analysis showing that keloid cases with a recurrent history have higher *PIEZO2* gene expression than do cases with non‐recurrence

**Figure 2:**
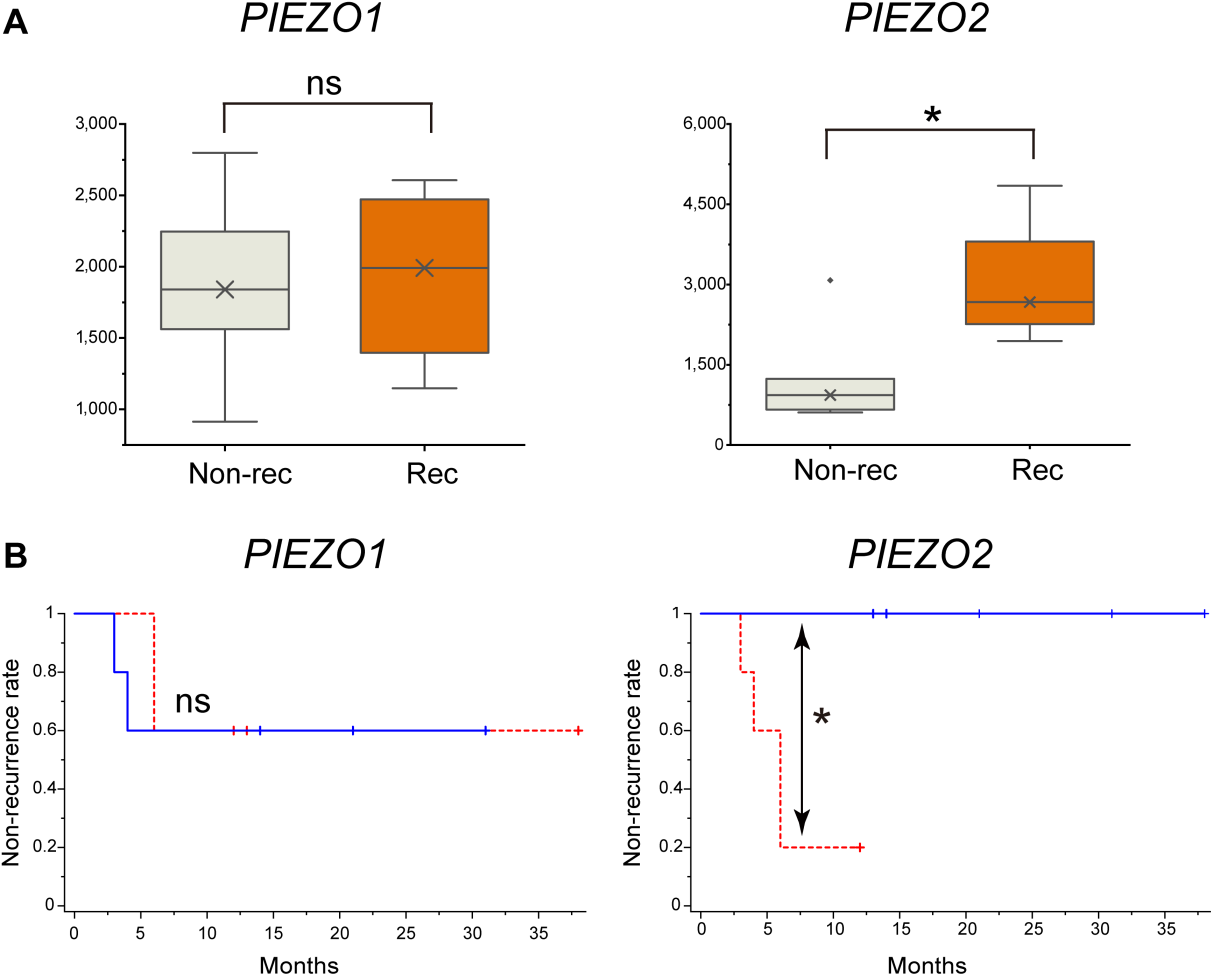
Expression level of the *PIEZO2* gene was higher in cases that recurred after keloid treatment than in cases that did not. (A, B) Analysis of the relationship between keloid recurrence after surgery and the expression levels of *PIEZO1*, *PIEZO2*, and other genes. (A) Comparison of *PIEZO2* expression levels in keloid recurrence cases (*n* = 4) and non‐recurrence cases (*n* = 6). The mean recurrence‐free follow‐up period in the non‐recurrence group was 21.5 ± 9.8 months. No significant difference was observed in *PIEZO1* expression levels between the two groups (1,934.3 ± 573.7 versus 1,866.8 ± 581.6, *p* = 0.88). However, a significant difference in *PIEZO2* expression levels was observed between the two groups (3,032.5 ± 1,090.2 versus 1,241.9 ± 820.7, *p* = 0.032). Rec: recurrence group; Non‐rec: non‐recurrence group. *p < 0.05. (B) Kaplan–Meier curve shows no difference in the recurrence rate between keloids with high *PIEZO1* expression levels (*n* = 5) and those with low *PIEZO1* expression levels (*n* = 5). Kaplan–Meier curves show that the frequency of post‐treatment recurrence differs between keloids with very high *PIEZO2* expression levels (*n* = 5) and those with moderately high *PIEZO2* expression levels (*n* = 5). The recurrence rate during the follow‐up period was significantly higher in the *PIEZO2* higher expression level group than in the *PIEZO2* lower expression level group (4/5 versus 0/5, *p* = 0.047). Red line: keloids with a significantly higher expression level of each gene; blue line: keloids with a lower expression level of each gene.

To investigate the effect of differences in *PIEZO2* expression on the trend to recur after surgical treatment, we evaluated *PIEZO2* expression levels in cases with and without recurrence. In a retrospective follow‐up study of keloid patients treated with a standardized protocol consisting of excision and adjuvant therapy, four cases showing signs of recurrence within 12 months and that required additional treatment, such as local steroid administration or re‐operation, were categorized as the recurrence group. These four cases showed significantly higher *PIEZO2* expression compared with non‐recurrent cases (Figure 2A). In contrast, no significant differences in the *PIEZO1*, *COL1A2*, and *POSTN* expression levels were observed between recurrent and non‐recurrent cases (Figure 2A and supplementary material, Figure S2). Additionally, there were no differences in background factors such as age, disease duration, clinical severity score, affected anatomical site, or treatment strategy between the two groups (Table 1).

**Table 1:** Comparison of the basic characteristics and expression levels of *PIEZO* genes between the recurrence and non‐recurrence groups.

	Recurrence group	Non‐recurrence group	*p* value
Number of cases	4	6	
Sex (F/M)	2/2	3/3	1.0
Age (years)	34.3 ± 14.5	30.3 ± 18.7	0.75
Post‐onset period (months)	41.3 ± 21.6	26.8 ± 6.6	0.37
JSS score	15.5 ± 2.7	11.8 ± 2.8	0.10
Location	Ear lobe (*n* = 1), chest (*n* = 2), abdomen (*n* = 1)	Ear lobe (*n* = 1), chest (*n* = 3), abdomen (*n* = 2)	
Radiation therapy (Y/N)	4/0	4/2	0.57
Relative expression level of *PIEZO1*	1,934.3 ± 573.7	1,680.5 ± 444.7	0.88
Relative expression level of *PIEZO2*	3,032.5 ± 1,090.2	1,241.9 ± 860.7	**0.032** *

*Note*: *p* value in bold is statistically significant.

* *p* < 0.05.

Kaplan–Meier curves showed a clear trend towards recurrence after surgical treatment. Indeed, the five cases with higher *PIEZO2* expression had a higher short‐term recurrence rate than did the cases with lower *PIEZO2* expression levels (*n* = 5), whereas a higher short‐term recurrence rate was not associated with higher *PIEZO1* expression (Figure 2B). Additionally, both *COL1A2* and *POSTN* expression showed a strong positive correlation with *PIEZO2* expression (Figure 1D and supplementary material, Figure S2). These findings suggest that the increased *PIEZO2* expression may be associated with a higher incidence of keloid recurrence following surgical treatment.

### Enhanced expression of *PIEZO2* in lymph‐endovascular cells and a subset of fibroblasts of the keloid tissue

**Figure 3:**
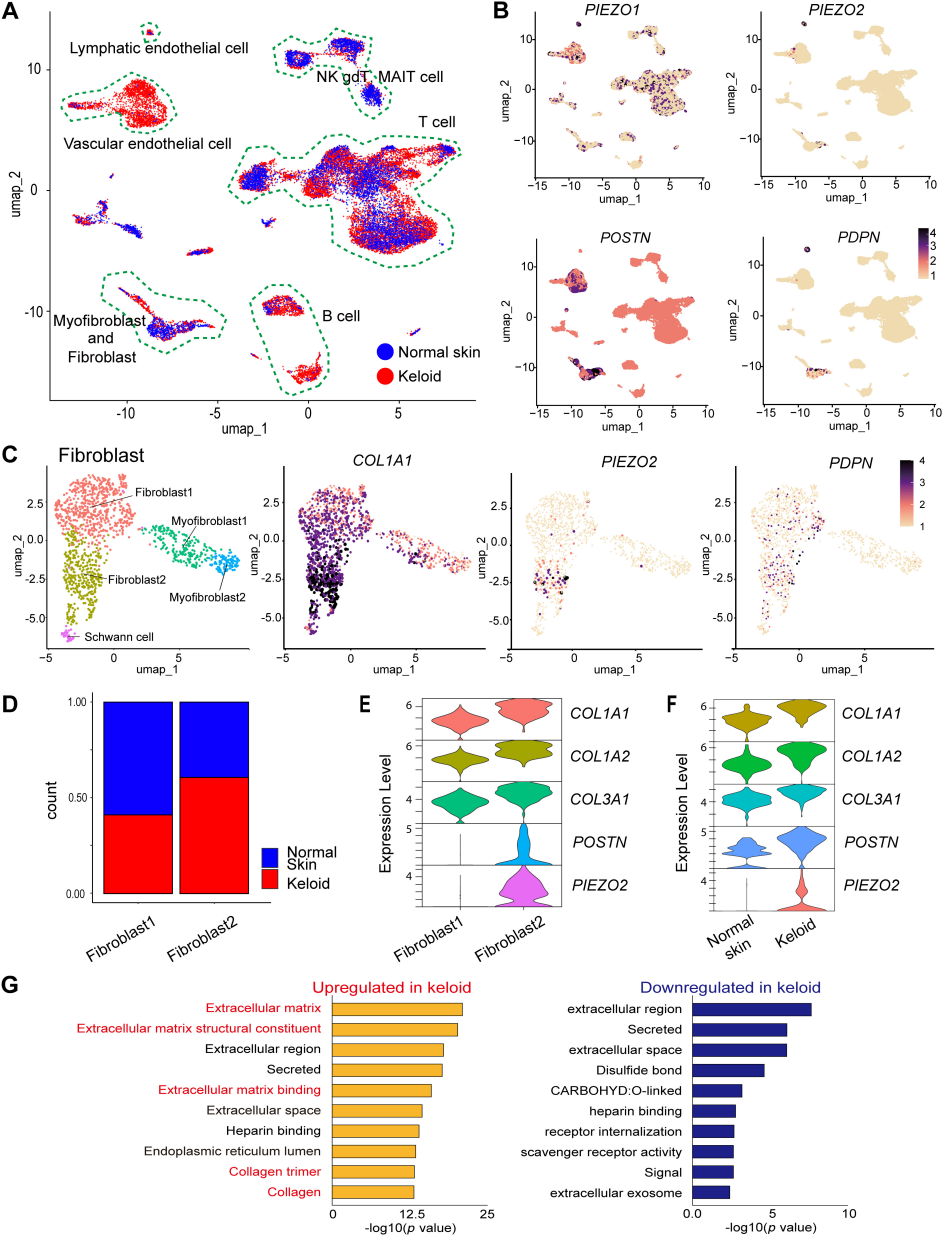
Enhanced expression of *PIEZO2* in lymph‐endovascular cells and a subset of fibroblasts of the keloid tissue. (A–G) Cells from keloid and normal skin tissues were isolated by FACS and analyzed by scRNA‐seq to search for genes characteristically expressed in keloids. (A) Normal skin and keloid cells were projected onto uniform manifold approximation and projection (UMAP) of scRNA‐seq libraries. The ratio of counts by cell cluster was compared between normal tissue and keloid tissue. Vascular endothelial cells and B cells were listed as having a higher proportion of expression in keloids than in normal skin. Blue dots: normal skin; red dots: keloid tissue. (B) Distribution of the expression of specific genes. *PIEZO1* was expressed in a wide variety of cells, whereas *PIEZO2* was expressed in fewer types of cells overall and was mainly expressed in VECs, LECs, and fibroblasts. *POSTN* was expressed in VECs and fibroblasts. *PDPN* was expressed in LECs and fibroblasts. (C) UMAP projections of fibroblasts, myofibroblasts, and Schwann cells were selected from the original dataset. Based on differences in expression characteristics, fibroblasts were classified as Fibroblast1 and Fibroblast2, and myofibroblasts as Myofibroblast1 and Myofibroblast2. Each cluster was color‐coded according to the cell subset. The expression levels of *COL1A1*, *PIEZO2*, and *POSTN* were significantly higher in Fibroblast2. (D) The ratio of Fibroblast1 to Fibroblast2 counts was compared between normal tissue and keloid tissue. The amount of expression was high in keloids for Fibroblast2. Blue bars: normal skin; red bars: keloid tissue. (E) The violin plot shows that *COL1A1*, *COL1A2*, *COL3A1*, *POSTN*, and *PIEZO2* are significantly more highly expressed in Fibroblast2 than in Fibroblast1. (F) The violin plot shows that the expression levels of *COL1A1*, *COL1A2*, *COL3A1*, *POSTN*, and *PIEZO2* in Fibroblast2 are greater in keloid tissue than in normal tissue. (G) Gene Ontology terminology analysis shows genes that are up‐ and down‐regulated in Fibroblast2 compared with normal skin, respectively. The expression of genes related to the extracellular matrix and collagen is upregulated.

Given the increased *PIEZO2* expression associated with recurrence of keloids in the short term, and the possible role of *PIEZO2*‐positive cells in the histopathological architecture of keloids, we performed scRNA‐seq analysis to characterize *PIEZO2*‐positive cells in keloid tissue. We performed gene expression analysis at the single‐cell level using cells gathered from three keloid skin tissues and one normal skin area (supplementary material, Table S2, cases 11–13), and by integrating the gene expression profiles, 26 clusters were identified unbiasedly, based on differentially expressed genes across a total of 10,000 cells (supplementary material, Figure S3A). Comparison between keloid and normal skin tissue showed that the primary increases occurred in immune cells, including T‐ and B‐cell clusters, and stromal cells, including vascular endothelial cells (VECs), lymphatic endothelial cells (LECs), and part of the fibroblast cell clusters (Figure 3A and supplementary material, Figure S3A,B). Notably, while most cells, including immune system cells, expressed molecules involved in fibrosis induction (*POSTN*, *TGFB1*, *AREG*), the key genes involved in fibrosis induction, *PDPN*, *FBN1*, *COL1A2*, *COL3A1*, *PLOD1*, and *PLOD2*, were mainly expressed in VECs, FCs, and LECs (Figure 3A and supplementary material, Figure S3C) [1, 26, 27, 28]. Additionally, while *PIEZO1* was expressed in most cells, including immune system cells, the expression of *PIEZO2* was limited to VECs, fibroblasts, and LECs. This suggests that the detected increase of *PIEZO2* expression in keloid cases prone to early recurrence may reflect the increased fibroblast, blood vessel, and collagen production in keloid tissue (Figure 3B).

Next, we analyzed the characteristic features of *PIEZO2*‐expressing cells that produced an extracellular matrix in keloids. Among fibroblasts, Schwann cells, and myofibroblasts, *PIEZO2* expression was exclusively confined to the Fibroblast2 cluster (Figure 3C). Notably, the Fibroblast2 cluster included cells with higher levels of *COL1A1* expression, and there were increased cell numbers in the Fibroblast2 cluster in keloids compared with normal tissue. Additionally, the Fibroblast2 cluster expressed higher levels of *COL1A1*, *COL1A2*, *COL3A1*, *PIEZO2*, and *POSTN* compared with the Fibroblast1 cluster (Figure 3D,E and supplementary material, Figure S3D,E). Moreover, the Fibroblast2 genes expressed were higher in keloids than in normal skin (Figure 3F and supplementary material, Figure S3F), and the increased magnitude of signaling related to increased extracellular matrix production (Figure 3G). Finally, results from CellChat analysis showed that outgoing signals from, and ingoing signals to, *PIEZO2*‐positive cells were involved in communication with stromal cells and immune cell populations (supplementary material, Figure S3G,J).

### *PIEZO2* ^hi^ fibroblasts in keloid tissue show enhanced expression of collagens and extracellular matrix

**Figure 4:**
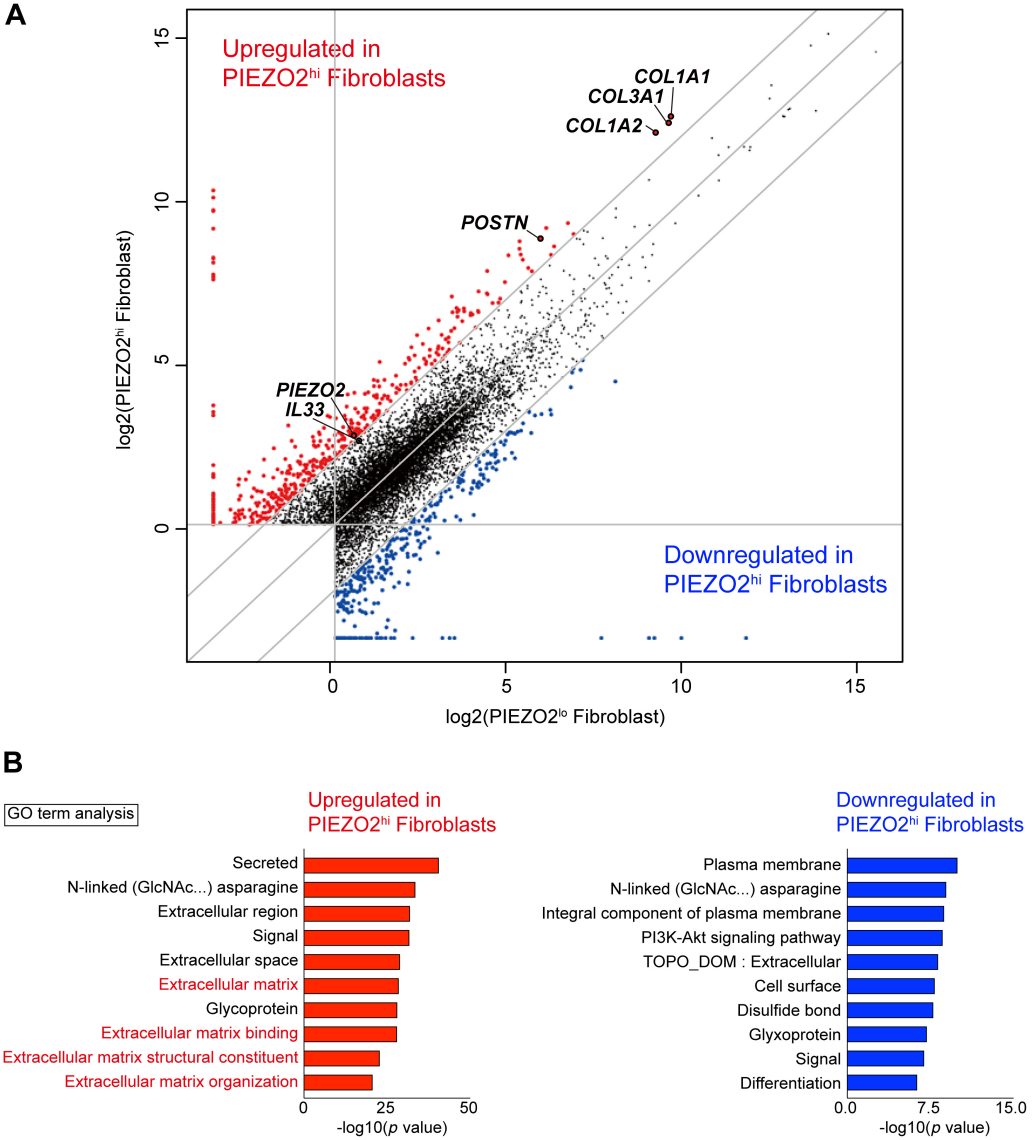
Fibroblasts with high *PIEZO2* expression levels in keloid tissue have high expression levels of a group of genes involved in tissue proliferation and *POSTN*. (A, B) Keloid tissue cells were separated by FACS, fibroblasts were sorted into *PIEZO2* high‐expressing and *PIEZO2* low‐expressing cells, and gene expression trends were analyzed. (A) Scatter plots show that the expression levels of various collagen genes, *POSTN*, and *IL33* are higher in *PIEZO2* high‐expressing fibroblasts. (B) The Gene Ontology term analysis shows genes up‐ and down‐regulated in *PIEZO2* high‐expressing fibroblasts versus *PIEZO2* low‐expressing fibroblasts, respectively. It was shown that the expression of genes related to the extracellular matrix was upregulated in *PIEZO2* high‐expressing fibroblasts.

To investigate the role of *PIEZO2*‐expressing fibroblasts in keloid tissue, we analyzed the global gene expression on fibroblasts isolated from keloid tissue (supplementary material, Table S2, case 14) from *PIEZO2* high‐expression (*PIEZO2* ^hi^) and *PIEZO2* low‐expression (*PIEZO2* ^lo^) groups. The scatter plot showed that *PIEZO2* ^hi^ fibroblasts expressed *COL1A1*, *COL1A2*, *COL3A1*, and *COL6A2* more highly than did *PIEZO2* ^lo^ fibroblasts (Figure 4A and supplementary material, Figure S4A). Gene Ontology (GO) term analysis showed upregulated extracellular matrix categories, including extracellular matrix structural constituent, and extracellular matrix organization, in *PIEZO2* ^hi^ fibroblasts (Figure 4B). However, the *PIEZO2* ^hi^ fibroblasts showed low expression levels of other markers of Merkel cells, suggesting a different cellular lineage from Merkel cells (supplementary material, Figure S4B). Notably, *PIEZO2* ^hi^ fibroblasts highly expressed *IL33* and *POSTN*, genes involved in the pathogenesis of itch in atopic dermatitis (Figure 4A), while they showed a relatively lower expression of genes associated with chemical itch than did *PIEZO2* ^lo^ fibroblasts (supplementary material, Figure S4C). These findings suggest that *PIEZO2* ^hi^ fibroblasts are involved in collagen production, and the expression of *IL33* and *POSTN*, but play a limited role in mediating chemical itch in keloids.

### PIEZO2‐expressing cells appear around the vessel structures in the dermis of keloid tissue

**Figure 5:**
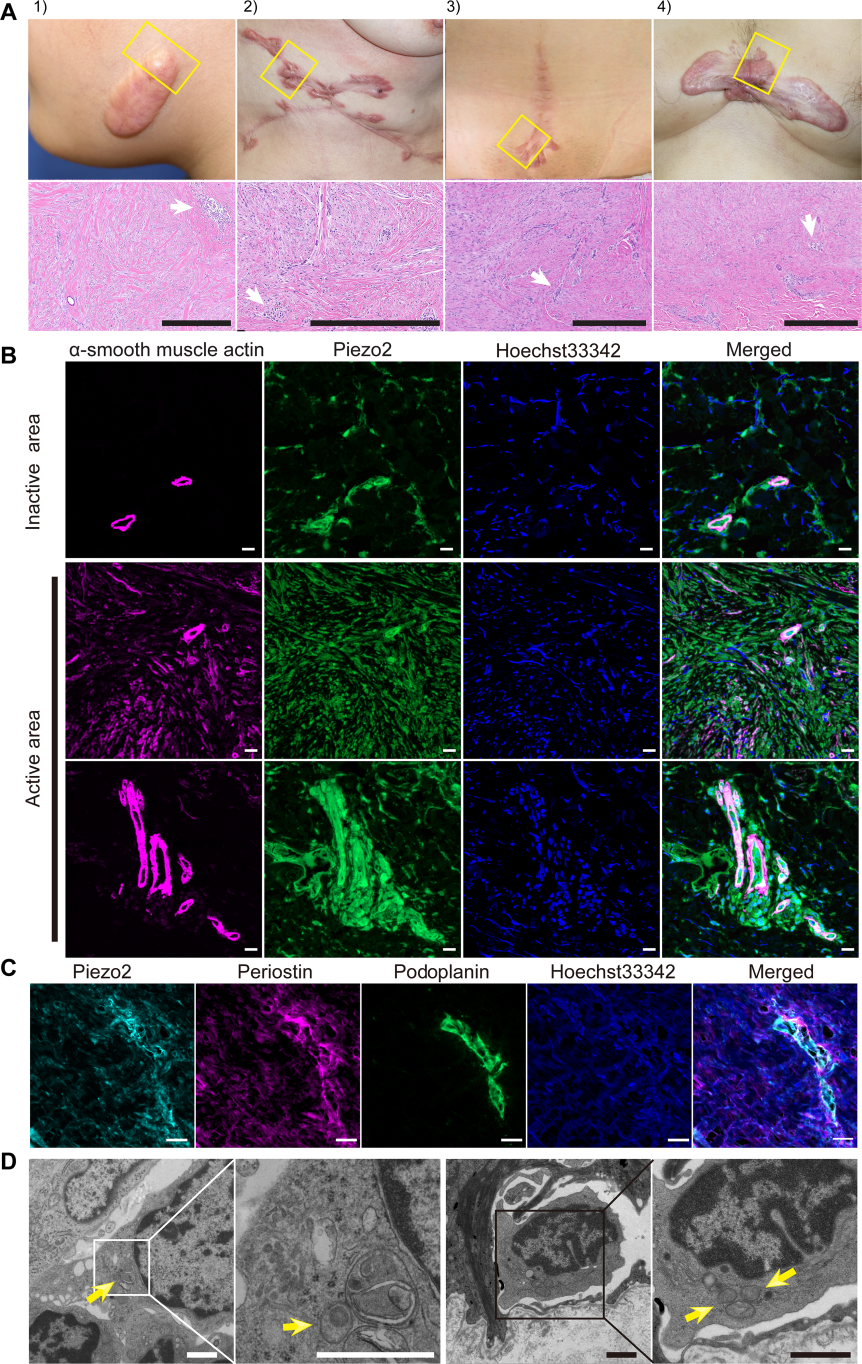
Small cells expressing PIEZO2 appear in the dermal layer of the keloid tissue. (A–C) Histological findings and (D) electron microscopy of small cell cluster in the dermal layer of the active growth area of keloid tissue. (A) Macroscopic appearance of keloid (upper row) and H&E staining (lower row). The square outlined in yellow in the upper row is the area for the specimens prepared from the excised keloid tissues for histological analysis. Written consent was obtained from all patients including for use of photographs. In the images of H&E staining, white arrowheads indicate areas where we identified small immune cells. Scale bars (black lines), 500 μm. Supplementary material, Figure S5 demonstrates loupe tissue images of the area of specimens prepared from each keloid case and the two microscopic tissue images at different magnifications, respectively. Cases, from left to right, are keloid case numbers 14, 24, 25, and 11 (see supplementary material, Table S2). (B) Representative confocal microscopy images of small cell populations in keloid dermal stained with anti‐α‐smooth muscle actin, anti‐PIEZO2, Hoechst 33342 (nucleus), and the merged image. Top: sample of a non‐active region of keloid. Middle and bottom: samples from active regions of keloid. In the proliferative areas, PIEZO2‐positive cells were densely distributed around the perivascular space and some co‐localized with immunohistochemically detected αSMA‐positive vascular structures. In contrast, in the inactive area, PIEZO2‐positive cells were sparse. Scale bars, 20 μm. (C) Representative confocal microscopy images of keloid skin microcell populations in areas of active proliferation, stained with anti‐PDPN, anti‐POSTN, anti‐PIEZO2, Hoechst 33342 (nucleus), and the merged images. Clusters of PIEZO2‐positive cells appeared around PDPN‐positive lymphatic vessels. Scale bars, 20 μm. (D) Representative transmission electron microscopy (TEM) images. Low‐power field TEM image (left) and high‐power field TEM image (second from left) of clustering cells in the dermal layer of keloid. Low‐power field TEM image (second from right) and high‐power field TEM image (right) of Merkel cells in the basal cell layer of the epidermis of the skin. Yellow arrows indicate dense core granules in the cytoplasm. Scale bars, 1 μm.

Next, we aimed to identify histopathological hallmarks of PIEZO2‐positive cells in keloid tissue by examining 16 excised keloid tissue cases. PIEZO2‐positive cells were present in all 16 keloid tissues, the distribution of which tended to be in the perivascular spaces around vessel structures in the dermis. PIEZO2‐positive cells were present in areas with small immune cells, including B cells and T cells, and tended to cluster in these regions (Figure 5A and supplementary material, Figure S5 and Table S6, cases 11–26). Notably, the clustering of PIEZO2‐positive cells with small immune cells was more pronounced in proliferative (active) areas of the keloid than in the central, more mature area (inactive) of the keloid (Figure 5B and supplementary material, Figure S6). Another hallmark of PIEZO2‐positive cells was the presence of both POSTN and PIEZO2‐positive cells, spreading from the lymphatic vessels into the dermal interstitium of keloid tissue (Figure 5C and supplementary material, Figure S7). Notably, lymphatic endothelial cells of these lymphatic vessels were positive for PDPN, POSTN, and PIEZO2. Additionally, TEM analysis revealed the presence of dense core granules in interstitial cells in the dermal peri‐lymphatic vessel space, similar to the dense core granules found in Merkel cells [29, 30] within the basal layer of the epidermis (Figure **5**D, indicated by yellow arrows). In summary, PIEZO2‐positive cells were present in the spaces around lymph vessels and vascular vessels, and TEM analysis confirmed the presence of high‐density granules within these PIEZO2‐positive cells.

## Discussion

**Figure 6:**
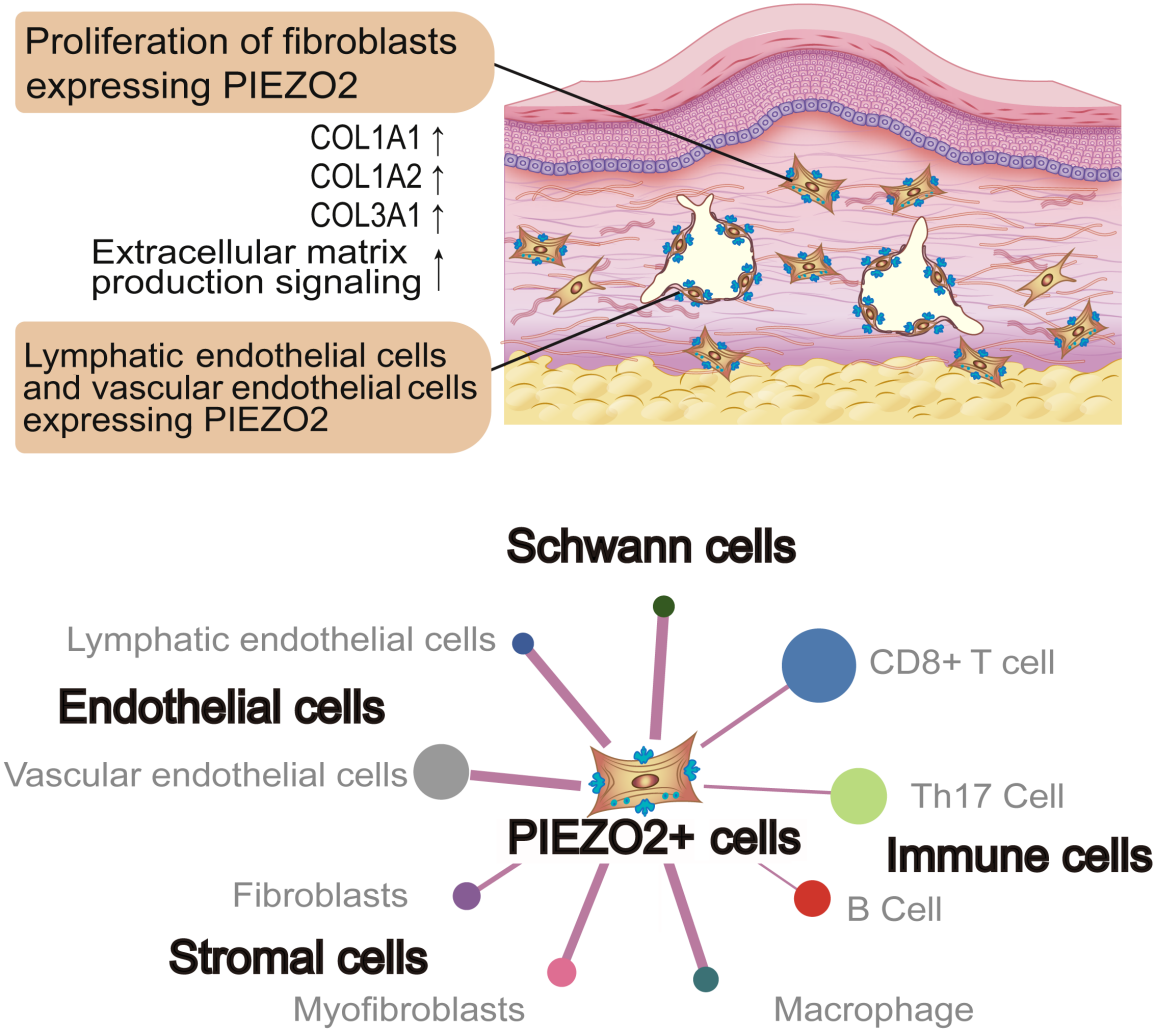
Schematic diagram proposing that cells expressing PIEZO2 mechanoreceptors are involved in the pathology of keloids. Fibroblasts, lymphatic endothelial cells, and vascular endothelial cells expressing PIEZO2 are involved in the pathology of keloids. In particular, fibroblasts expressing PIEZO2 show signaling activation that promotes extracellular matrix production. PIEZO2‐positive cells form a dense network of intercellular interactions with various cells, including stromal cells, endothelial cells, Schwann cells, and immune cells. The figure reflects the node size and edge width in the analysis results presented in this study.

In this study, we report increased expression of *PIEZO2* in fibroproliferative keloid tissue and elucidate that PIEZO2‐positive cells are involved in the formation of keloid vessel structures and fibrous stroma as a pathological scar (Figure 6). As recurrence episodes following surgical excision of fibroproliferative keloid tissue were associated with higher expression of *PIEZO2*, we focused on *PIEZO2* and found that *PIEZO2*‐positive VECs, LECs, and fibroblasts existed in keloids. Notably, *PIEZO2*‐positive fibroblasts were identified as a new subpopulation of fibroblasts, appearing in fibroproliferative tissue expressing higher collagen genes than typical fibroblasts. Moreover, as we did not observe induction of *PIEZO2* expression in lymphedema cases, where fibroproliferation occurs without mechanical tension influencing disease progression, we propose a concept where keloid tissue is a pathological scar coordinately constructed by PIEZO2‐positive cells and inflammatory cells.

We propose that the appearance of PIEZO2‐positive cells in the fibroproliferative tissue of keloids is a unique finding concerning keloid pathology for the following reasons. First, the appearance of PIEZO2‐positive cells in the dermis is ectopic. Indeed, PIEZO2 is expressed on Merkel cells in the epidermal basal layer in normal skin [16, 29, 30, 31, 32, 33, 34], the sensing role of which is carried in the epidermal layer to send the signals to Aβ afferent nerve fibers, by changing the nerve‐ending membrane potential near the epidermis [35, 36, 37]. Therefore, PIEZO2‐positive cells including VECs, LECs, and fibroblasts in the dermis may ectopically stimulate afferent nerve fibers. Second, scRNA‐seq analysis characterized *PIEZO2*‐positive fibroblasts as a previously unknown subset of fibroblasts, with distinct gene expression profiles. Indeed, *PIEZO2*‐positive fibroblasts, compared with other typical fibroblasts, enhanced gene expression to drive the production of collagen fibers and substances in the extracellular matrix. Third, the elevated *PIEZO2* expression in keloid tissues was associated with a higher risk of early recurrence following resection. Concerning this finding, VECs, LECs, and fibroblasts spreading around the vascular structures in keloid tissue expressed *PIEZO2*, contributing to increased *PIEZO2* expression in the fibroproliferative tissue of keloids. For these reasons, we considered the appearance of *PIEZO2*‐positive fibroblasts as the primary keloid pathology.

For the following reasons, we also consider that *PIEZO2*‐positive cells in the fibroproliferative tissue of keloids should be distinct from Merkel cells in the epidermis. First, results from scRNA‐seq analysis classified *PIEZO2*‐positive cells into endothelial cells in lymphatic and blood vessels and fibroblast subsets but differentiated them from Merkel cells that have keratinocyte properties [29, 38]. Second, while previous studies have reported that Merkel cells are present in the dermis of fetuses, newborns, and sometimes even adults [31, 39, 40], Merkel cells are generally keratinocyte lineage cells located in the basal layer of the epidermis in adults, after the fetal and neonatal periods [29]. However, *PIEZO2*‐positive cells in the fibroproliferative tissue of keloids should be distinguished from the ‘dermal Merkel cell’ used in earlier studies. The development and differentiation of PIEZO2‐positive cells that construct the vascular structure and fibrous stroma in keloids are still unclear. Therefore, further studies are needed to investigate the influence of keloid‐related PIEZO2‐positive cells on morphological diversity in keloid tissue during the differentiation and maturation process.

The vascular structure and fibrous stroma of keloids containing PIEZO2‐positive cells indicated that chronic tension may activate the PIEZO2 receptor, contributing to the abnormal growth of scar tissue (Figure 6). This finding provides further insight into the function of the PIEZO receptor, as earlier reports demonstrated that mechanical stimulation of cells with overexpression of the PIEZO1 receptor can promote scar tissue growth [13, 14], and calcium ions entering through the activated PIEZO2 induced the activation of intracellular signaling pathways [11, 41]. Additionally, our results show that intercellular communication between PIEZO2‐positive cells and immune cells may contribute to the pathology of keloids (supplementary material, Figure S3G,J). Therefore, our findings on PIEZO2‐positive cells support the hypotheses that mechanical tension and persistent inflammation contribute to the pathogenesis of keloids [9, 10, 42, 43]. At the same time, respecting earlier studies reporting no increment in inflammation‐related genes in keloid tissue [1, 44], careful case selection is essential to assess the contribution of immune cells. Indeed, since our results are from classified keloid cases with a high‐to‐moderate recurrence risk based on JSS scores [18, 19], we suggest that further keloid studies use the JSS score to investigate the molecular mechanisms.

We consider Fibroblast2 a new subset group in fibroblasts as there are no previous reports on *PIEZO2* expression on fibroblasts. In this study, we showed exclusive *PIEZO2* expression in Fibroblast2, with lower expression of *PIEZO2* in Fibroblast1, myoFibroblast1, and myoFibroblast2 (Figure 3C). So far, four subgroups of fibroblasts have been identified, including inflammatory, mesenchymal, papillary, and reticular fibroblasts, and we considered Fibroblast1 as the inflammatory fibroblast due to the high expression of APOE [1]. However, Fibroblast2 was challenging to classify into one of the four subsets since it did not express any of the typical differentiation markers for these four subsets. It is possible to classify Fibroblast2 as a mesenchymal fibroblast based on *POSTN‐*high expression. Indeed, the upregulated *PIEZO2* gene expression in keloids aligns with earlier studies demonstrating higher expression of *COL1A2* and *POSTN* in keloids than in normal skin tissue [1]. However, Fibroblast2 is a cell that appears in pathological scar tissue with a high risk of recurrence. Additionally, the upregulation of collagen gene expression in Fibroblast2 and the activation of signaling pathways for ECM production (Figures 3E,G and 4B) may support the idea that *PIEZO2* expression contributes to the characteristic histological features in abnormally proliferating scar tissue in keloids. Therefore, we discuss Fibroblast2 as a new subset in this study.

Concerning developing dysesthesia in keloids, since PIEZO2 functions to convert the mechanical touch into electrical signals stimulating the distributing nerve endings in the dermis, PIEZO2‐positive cells forming the vascular structure and fibrous stroma in keloids may contribute to sensory abnormalities [35, 37, 45]. Earlier studies have indicated that the activation of PIEZO2 in Merkel cells may trigger sensory abnormalities in keloids [46, 47, 48]. However, these studies did not consider the involvement of VECs or LECs.

The study has several limitations. It is based on a small case series and was conducted in a relatively homogeneous Asian population. Additionally, we did not examine the mechanical pressure sensed by PIEZO2‐positive cells or investigate downstream intercellular signaling cascades induced by mechanical stretch. These limitations will be addressed in future studies.

Targeting PIEZO2 may be effective in the treatment of keloids, as earlier studies pointed out that proliferation of fibroblasts and myofibroblasts in response to mechanical stimulation through PIEZO1 could serve as a therapeutic target [49, 50]. In this context, our findings on PIEZO2 expression in keloid tissue open new avenues for research aimed at elucidating the functional activation of the PIEZO2 receptor and evaluating the effects of its inhibition or remission of keloid tissue using molecular and cellular biology techniques. In line with developing novel keloid treatments, we have initiated a clinical study focusing on PIEZO2 expression to establish unique molecular signatures (biomarkers) for monitoring the therapeutic efficacy of PIEZO2‐targeted interventions.

## Author contributions statement

TN and YuI conceived and designed the study. SA, NM, KA, SO, JTK, TN and KH provided clinical expertise and performed sample acquisition and administrative coordination. SA, NM, YH and JTK provided clinical expertise, obtained patient consent, and performed sample acquisition. SI, JTK, KA, SO and YuI performed the histological analysis and pathological review of samples. YA, SI and YuI performed the electron microscopy analysis. SI and YuI performed RNA‐seq analysis, and MK, SA, HM, KK, TN and KH performed scRNA‐seq experiments and computational data analysis, showing the characteristics and potential of *Piezo2*‐expressing fibroblasts. YoI and HH conducted the statistical analyses of clinical data. SA, SI, KH, TN and YuI contributed to the data interpretation. SA, SI, NM, KH and YuI wrote the manuscript.

## Supporting information


Supplementary materials and methods
**Figure S1**. Histological analysis using hematoxylin and eosin (H&E) staining and multiplex immunofluorescence staining on serial sections to visualize the spatial distribution of PIEZO2‐positive cells
**Figure S2**. Analysis of the relationship between *POSTN* or *COL1A2* gene expression levels and keloid recurrence
**Figure S3**. Enhanced expression of *PIEZO2* in lymphatic endovascular cells and a subset of fibroblasts within keloid tissue
**Figure S4**. Comparisons of gene expression trends between *PIEZO2* ^hi^ and *PIEZO2* ^lo^ cells
**Figure S5**. Hematoxylin and eosin staining showing the histological appearance of specimens prepared from excised keloids
**Figure S6**. Histological analysis using H&E staining and merged images from multiplex immunofluorescence staining showing PIEZO2‐positive cells
**Figure S7**. Spatial distribution of PIEZO2, periostin, and podoplanin in keloid tissue
**Figure S8**. Quality control and expression of marker genes for each cluster in single‐cell RNA sequencing (scRNA‐seq) (referred to in Supplementary materials and methods)
**Table S1**. Details of patients with keloids (cases KL1–KL10), severe lymphedema (SL1–SL10), and mild lymphedema (ML1–ML10) used for gene expression level comparison
**Table S2**. Details of patients with keloids (cases 11–26)
**Table S3**. Relative expression ratios of RNA in tissues from patients with mild lymphedema (ML), severe lymphedema (SL), and keloids
**Table S4**. Results of Kruskal–Wallis test comparing the mild lymphedema, severe lymphedema, and keloid groups
**Table S5**. Results of the Wilcoxon test comparing gene expression between groups
**Table S6**. Results of Pearson's correlation coefficient test between two genes based on relative RNA expression ratios (*n* = 30)

## Data Availability

The data that support the findings of this study have been deposited in NCBI's Gene Expression Omnibus and are accessible through GEO Series accession numbers GSE266334 (https://www.ncbi.nlm.nih.gov/geo/query/acc.cgi?acc=GSE266334), GSE266338 (https://www.ncbi.nlm.nih.gov/geo/query/acc.cgi?acc=GSE266338), and GSE274709 (https://www.ncbi.nlm.nih.gov/geo/query/acc.cgi?acc=GSE274709). Any additional information required to reanalyze the data reported in this paper is available from the corresponding author upon reasonable request.
