# Supplementary material for: Increased expression of the PIEZO2 mechanoreceptor in fibroblasts and endothelial cells within the lymphatic and vascular vessels of keloids
Source: J Pathol. 2025 Jul 31;267(1):105–19. doi: 10.1002/path.6455 (PMC12337814; doi:10.1002/path.6455)
Supplement: Supplementary file 1 — Supplementary materials and methods Figure S1. Histological analysis using hematoxylin and eosin (H&E) staining and multiplex immunofluorescence staining on serial sections to visualize the spatial distribution of PIEZO2‐positive cells Figure S2. Analysis of the relationship between POSTN or COL1A2 gene expression levels and keloid recurrence Figure S3. Enhanced expression of PIEZO2 in lymphatic endovascular cells and a subset of fibroblasts within keloid tissue Figure S4. Comparisons of gene expression trends between PIEZO2 hi and PIEZO2 lo cells Figure S5. Hematoxylin and eosin staining showing the histological appearance of specimens prepared from excised keloids Figure S6. Histological analysis using H&E staining and merged images from multiplex immunofluorescence staining showing PIEZO2‐positive cells Figure S7. Spatial distribution of PIEZO2, periostin, and podoplanin in keloid tissue Figure S8. Quality control and expression of marker genes for each cluster in single‐cell RNA sequencing (scRNA‐seq) (referred to in Supplementary materials and methods) Table S1. Details of patients with keloids (cases KL1–KL10), severe lymphedema (SL1–SL10), and mild lymphedema (ML1–ML10) used for gene expression level comparison Table S2. Details of patients with keloids (cases 11–26) Table S3. Relative expression ratios of RNA in tissues from patients with mild lymphedema (ML), severe lymphedema (SL), and keloids Table S4. Results of Kruskal–Wallis test comparing the mild lymphedema, severe lymphedema, and keloid groups Table S5. Results of the Wilcoxon test comparing gene expression between groups Table S6. Results of Pearson's correlation coefficient test between two genes based on relative RNA expression ratios (n = 30) [file PATH-267-105-s001.docx]

**Increased expression of the** **PIEZO2 mechanoreceptor in fibroblasts and endothelial cells within the lymphatic and vascular vessels of keloids**

S Akita, S Ikehara *et al. J Pathol* <https://doi.org/10.1002/path.6455>

**Supplementary materials and methods**

**Supplementary Figures S1–S8**

**Supplementary Tables S1–S6**

Reference numbers refer to the main text list

**Supplementary materials and methods**

*RNA sequencing for the clinical characteristics and prognosis of fibrous diseases*

Total RNA purification was performed using an RNeasy Plus Kit (QIAGEN, Venlo, The Netherlands) with an RNase-free DNase Set (QIAGEN) following the manufacturer’s protocol and as described in our previous study [51]. In brief, twenty µm-thick sections were cut from cryopreserved skin tissue using a cryostat and dissolved in Lysis Buffer in the RNeasy Kit (QIAGEN) to extract total RNA. The quality and amount of RNA in each sample were evaluated using Experion (Bio-Rad, Hercules, CA, USA) and NanoDrop (Thermo Fisher Scientific, Waltham, MA, USA), respectively. For RNA-seq analysis using a next-generation sequencer (NGS), library construction and sequencing for the Illumina HiSeq 4000 (Illumina, San Diego, CA, USA) were provided as a custom service of Eurofins Genomics K.K. (Tokyo, Japan). PolyA fraction (mRNA) was isolated from total RNA, followed by its fragmentation. Double-stranded (ds) cDNA was subsequently reverse-transcribed from fragmented mRNA. The ds cDNA fragments were processed for adaptor ligation, size selection (for 200-bp inserts), and amplified to generate strand-specific cDNA libraries. Prepared libraries were subjected to paired-end 2 × 101 bp sequencing on the HiSeq 4000 platform (Illumina) using the HiSeq 3000/4000 SBS Kit (Illumina) [52,53].

*Preparation of single-cell suspensions from the resected skin tissue and scRNA-seq*

Single-cell suspensions were prepared from the resected skin tissue. Keloid tissues were dissociated in a gentle MACS C-tube (Miltenyi Biotec, Bergisch Gladbach, Germany) with digestion buffer composed of Dulbecco’s Modified Eagle’s Medium (Gibco, Waltham, MA, US) and a Multi Tissue Dissociation Kit 1 using a gentle MACS Octo dissociator (Miltenyi Biotec). After running the program ‘37 °C_multi_H’, cell suspensions were passed through a 70-μm nylon mesh sheet. Libraries were prepared using Chromium Single Cell 3' Reagent Kits v3.1 following the manufacturer’s protocol (10× Genomics, Pleasanton, CA, USA). The generated scRNA-seq libraries were sequenced using a total of 127 cycles (paired-end reads) using a NovaSeq 6000 (Illumina).

Sequencing reads were processed and aggregated using Cell Ranger v6.0 (10× Genomics). Cells with nFeature RNA < 1,000 or > 4,000 detected genes or > 10% mitochondrial gene expression were excluded. Data were log-normalized using NormalizeData, and highly variable genes were identified using *FindVariableFeatures* (supplementary material, Figure S4A). We regressed out total UMI (unique molecular identifier) counts per cell, performed PCA (principal component analysis), and significant PCs (principal components) were selected based on the elbow plot. Clustering was performed using *FindNeighbors* and *FindClusters* (resolution = 2.0), and UMAP (uniform manifold approximation and projection) embedding was generated using *RunUMAP* (supplementary material, Figure S8B).

*Analysis and graphical display of RNA-seq data from fibrous diseases patients*

A total of 9,769 genes in which the maximum FPKM value of each gene was R1.0 were included in further analyses. Pathway analysis was performed using DAVID (https://david.ncifcrf.gov/tools.jsp, accessed 30 October 2024) [54,55] based on the GO term database (https://geneontology.org/, accessed 30 October 2024) [56,57]. A scatter plot was constructed using the R software program (The R Foundation for Statistical Computing, Vienna, Austria). The group of genes (the 21 ligands and ligand synthases) involved in itch was reanalyzed and selected from a previously reported dataset (GEO: GSE114921). For expression profiling among lymphedema and keloids, raw read counts were created using SAMtools (version 1.6) (https://samtools.sourceforge.net/, accessed 19 July 2022) [58]. Using the information of Gene ID, read counts for each transcript were summarized into those for each gene. Normalization of gene expression was performed using the following procedure [59,60]: (1) Total count normalization was performed, based on the following calculation. (2) A Trimmed Mean of *M*-values (TMM) normalization and differential expression analysis were performed using the edgeR (version 3.16.5) package (https://bioconductor.org/packages/3.21/bioc/html/edgeR.html, accessed 19 July 2022) [59]. Gene annotations were added to the results, using our in-house script.

*Cell–cell communication analysis and graphic display of scRNA-seq data from keloid cells*

We applied the R package CellChat (version 1.6.1) (<https://github.com/sqjin/CellChat>, accessed 16 April 2025) [23,61,62] to analyze the cell–cell communication networks within our scRNA-seq data, by which we assessed the expression levels of receptors and ligands to infer intercellular communication linked with the cells expressing *PIEZO2*. Based on the normalized expression matrix, we defined that cells expressing *PIEZO2* as for the gene expression level of *PIEZO2* exceeded a threshold value of 1. We employed the functions computeCommunProb and computeCommunProbPathway to evaluate the communication probability of ligand–receptor pairs and the associated signal transduction pathways. Subsequently, we visualized the strength of intercellular communication networks between *PIEZO2*-positive cell clusters and other cell-type clusters using the netVisual_circle function (supplementary material, Figure S3G–H). Furthermore, we confirmed the significant ligand–receptor interactions between *PIEZO2*-positive cell clusters and other cell-type clusters using the netVisual_bubble function (data not shown).


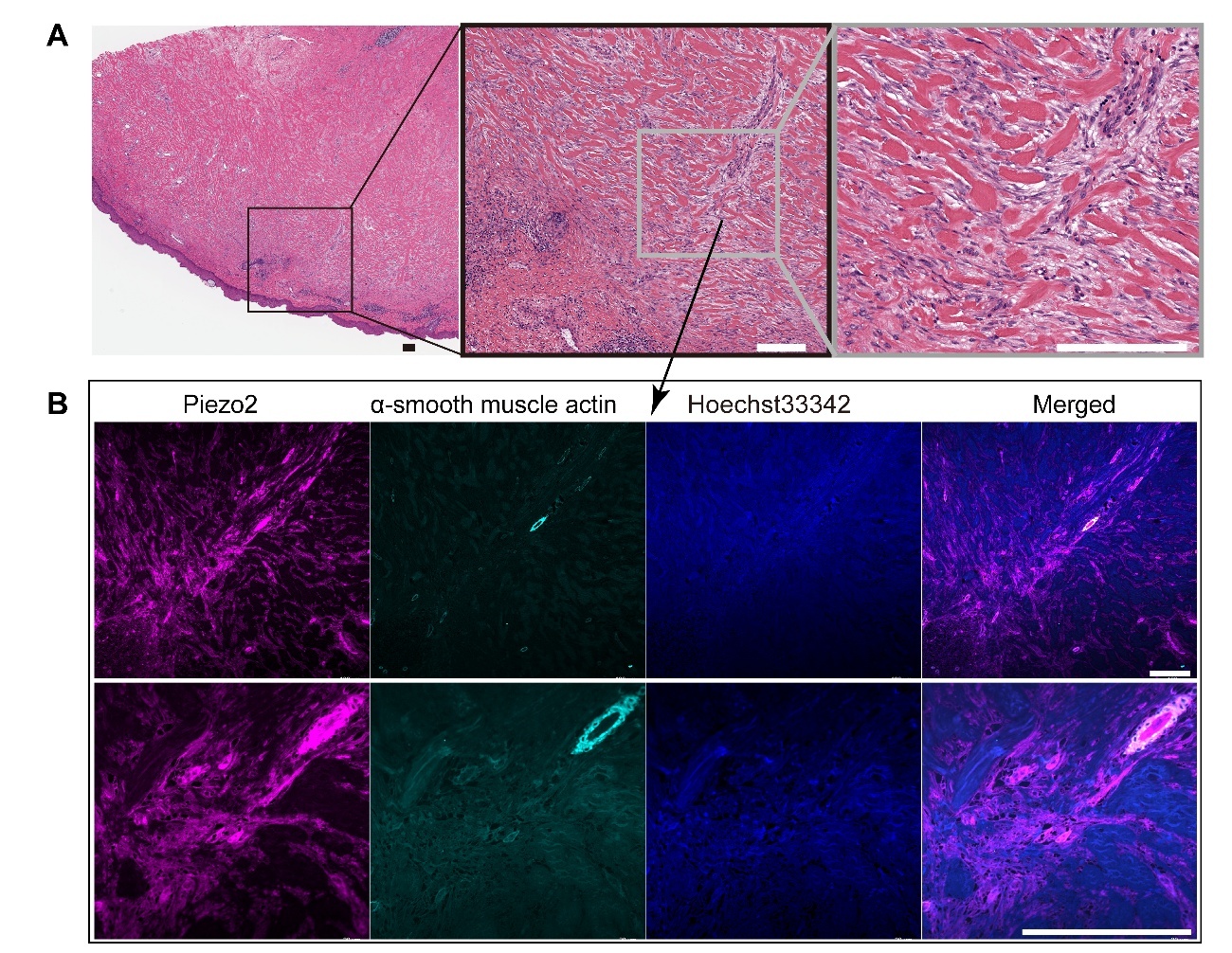


**Figure S1.** **Histological analysis using hematoxylin and eosin (H&E) staining and multiplex immunofluorescence staining on serial sections to visualize the spatial distribution of PIEZO2-positive cells.** Supplementary analysis supporting the main findings shown in Figure 1E. (A) H&E staining shows characteristic fibro-collagenous proliferation in the dermis as well as densely clustered inflammatory cells in both the dermis and the subcutaneous tissue. Vascular structures extending from the dermis to the subcutaneous tissue are apparent in the middle image (black-lined rectangle frame). In the image to the right, small and bland-spindle-shaped cells are distributed in the widened (or separated) space between haphazardly arranged thick collagen fibers in the dermis (gray-lined rectangle frame). Scale bars (black and white lines): 200 µm. (B) Multiple fluorescent immunofluorescence staining demonstrated PIEZO2-positive (magenta) cells. PIEZO2-positive cells are distributed on and around α-smooth muscle actin (αSMA: cyan)-positive cells. Hoechst 33342 staining visualizes nuclear structures. Scale bars (white lines): 200 µm.


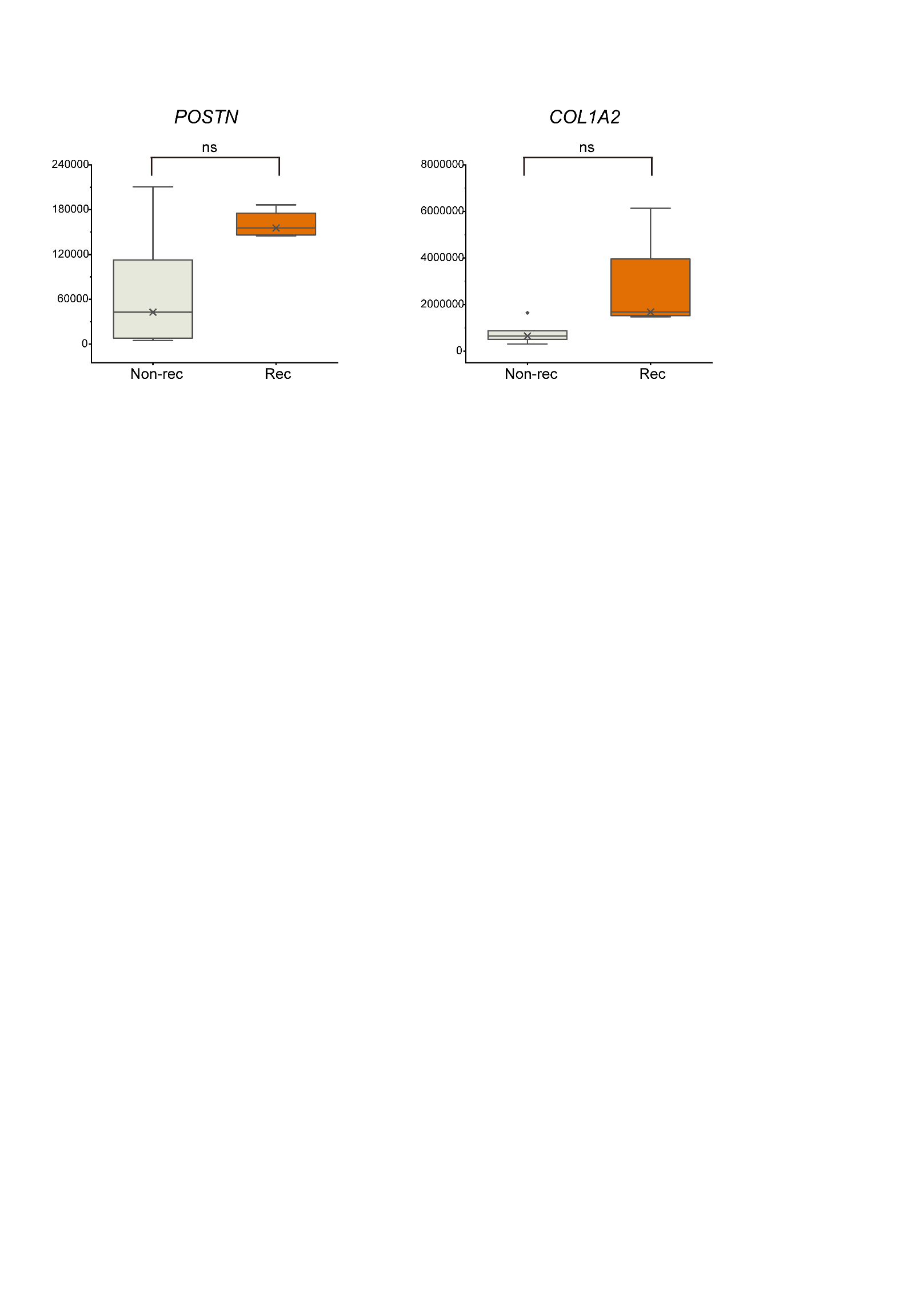


**Figure S2. Analysis of the relationship between *POSTN* or *COL1A2*** **gene expression levels and keloid recurrence.** Supplementary analysis results supporting the main findings shown in Figure 2. No significant differences in *POSTN* or *COL1A2* expression levels were observed between the recurrence group and the non-recurrence group.


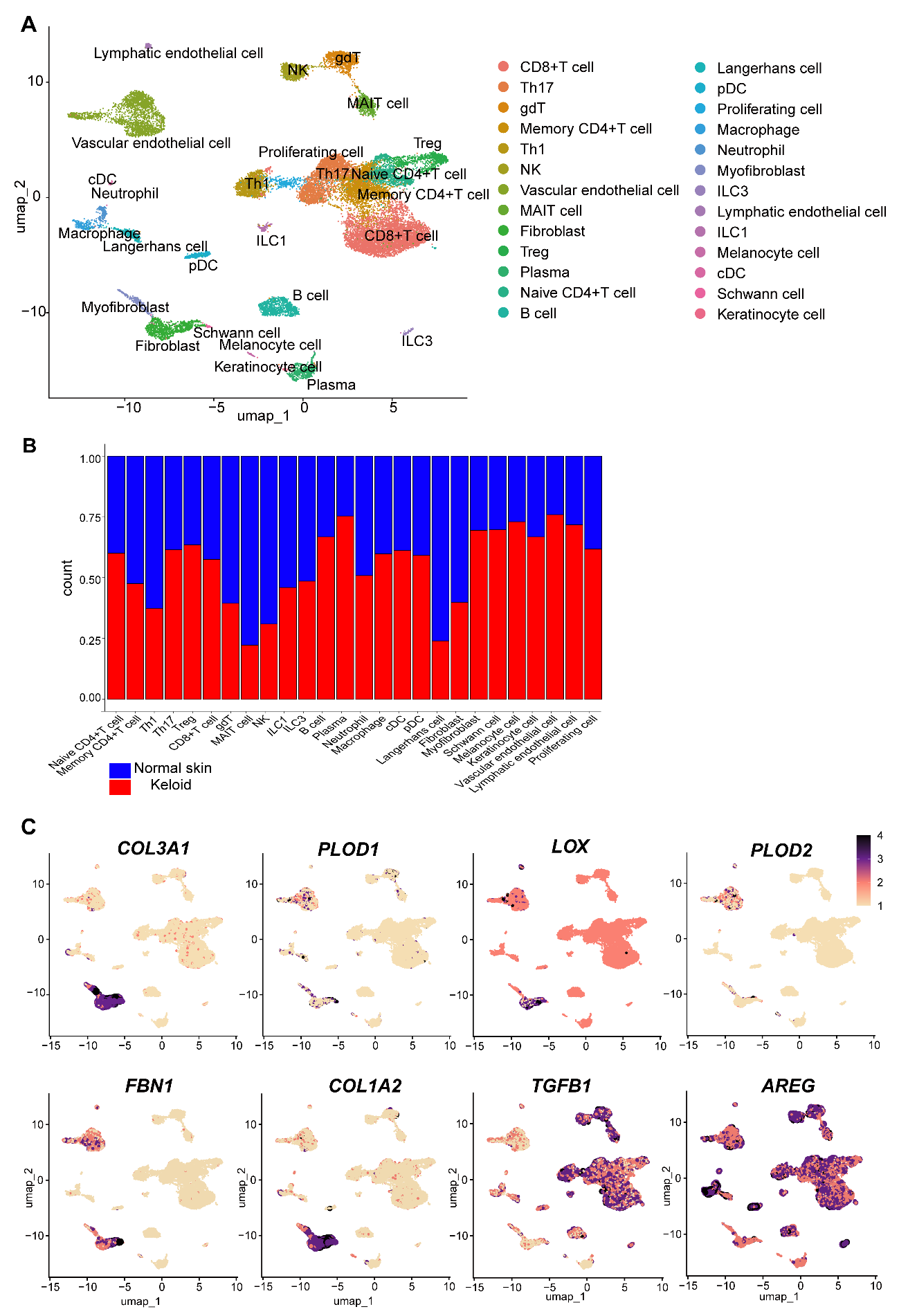


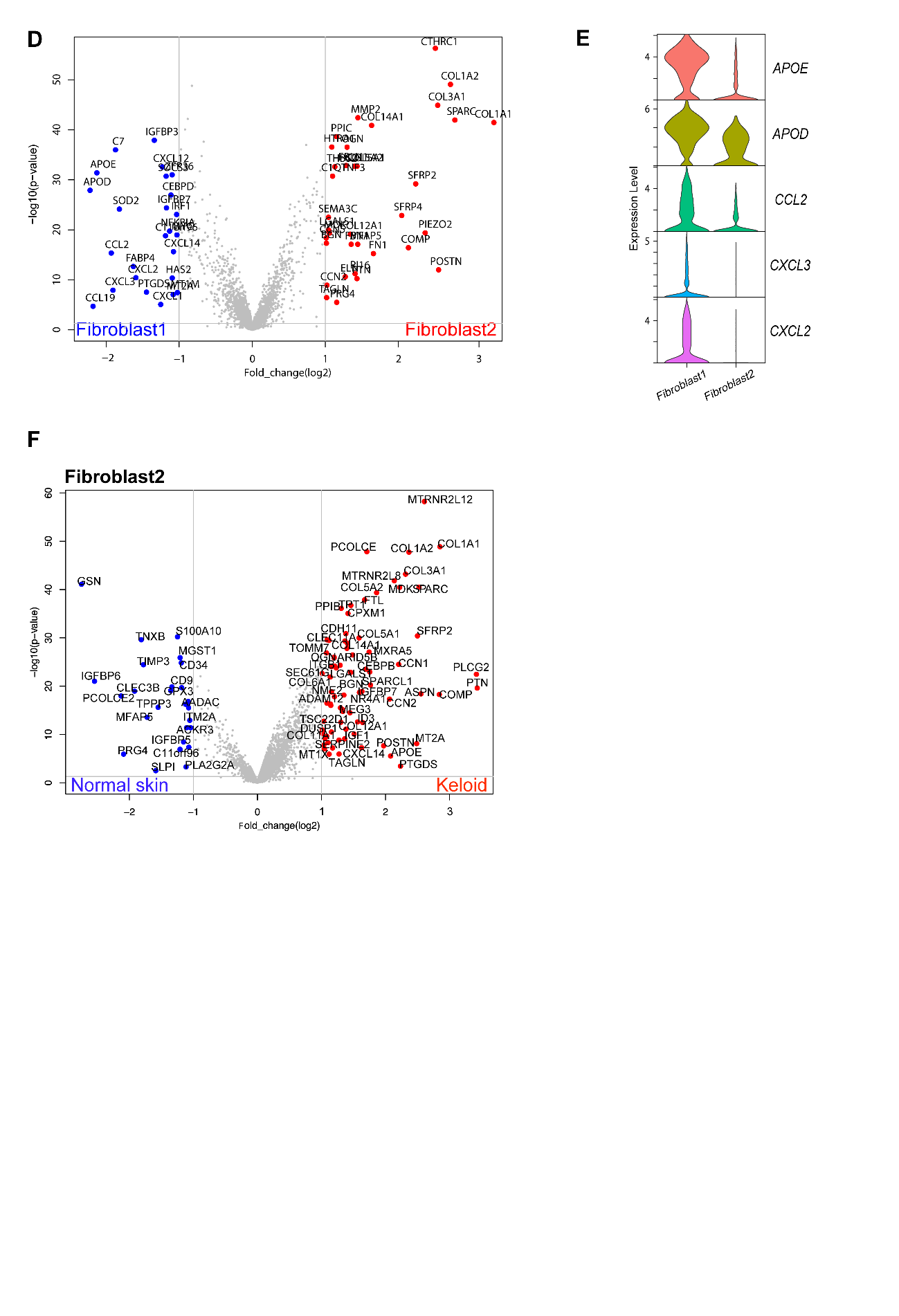


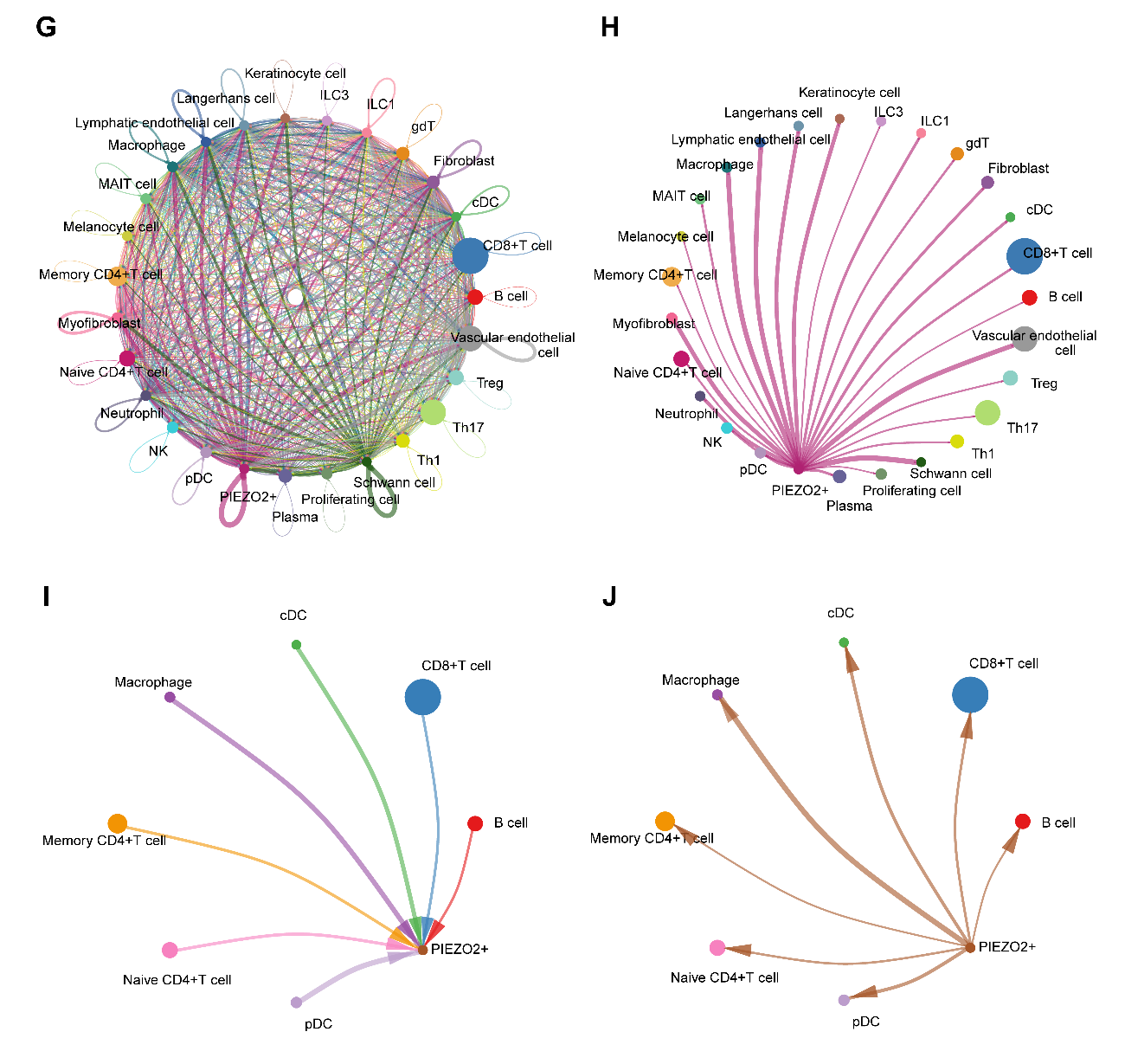


**Figure S3. Enhanced expression of *PIEZO2* in lymphatic endovascular cells and a subset of fibroblasts within keloid tissue.** (A–F) Supplementary results supporting the main findings presented in Figure 3. (A) Normal skin and keloid cells were color-coded and projected onto UMAP of scRNA-seq libraries. (B) The ratio of counts by cell cluster was compared between normal tissue and keloid tissue. Blue bars: normal skin; red bars: keloid tissue. (C) Distributions of *COL3A1*, *PLOD1*, *LOX*, *PLOD2*, *FBN1*, *COL1A2*, *TGFB1*, and *AREG* expression. (D) Volcano plot showing the distribution of genes that differ in expression levels between Fibroblast1 and Fibroblast2. (E) Violin plot showing significantly higher expression of *APOE*, *APOD*, *CCL2*, *CCL3*, and *CXCL2* in Fibroblast1 than in Fibroblast2. (F) Volcano plot showing the comparison of gene expression levels that differ between keloids and normal skin in Fibroblast2. (G) Through ligand–receptor interactions, *PIEZO2*-positive cells with keloids send and receive intercellular communication signals with immune cells and stromal cells through ligand–receptor interactions. This circle plot illustrates the intercellular interactions between *PIEZO2*-positive cells and other cell types in the keloid group. The size of each cell cluster node represents the number of cells, while the width of the edges corresponds to the number of ligand–receptor pairs involved in these interactions. (H) Outgoing signals from *PIEZO2*-positive cells communicate not only with stromal cells, including endothelial cells and Schwann cells, but also with immune cells, such as CD8^+^ T cells, Th17, and regulatory T cells (Tregs). This circle plot represents the network analysis results of signals originating from *PIEZO2*-positive cells. The findings suggest that *PIEZO2*-positive cells, capable of detecting mechanical stress, play a significant role in promoting the proliferation, activation, and diverse responses of these cells within keloids. (I) CellChat analysis results show a network through which *PIEZO2*-positive cells receive signals from immune cells, suggesting potential abnormalities in regulating or suppressing immune responses mediated by these cells. This circle plot illustrates the intercellular communication between immune cells and *PIEZO2*-positive cells that receive signals from them in the keloid group. The width of each edge indicates the number of ligand–receptor pairs. *PIEZO2*-positive cells receive ligand stimulation from various immune cells, including CD8⁺ T cells, macrophages, and conventional dendritic cells (cDCs). Notably, macrophages send the most significant signals to *PIEZO2*-positive cells. Additionally, signals from memory-type CD4⁺ T cells, undifferentiated CD4⁺ T cells, plasmacytoid dendritic cells (pDCs), and B cells transmit to *PIEZO2*-positive cells at a moderate to mild level. These findings suggest that the immune microenvironment of keloids has a considerable influence on the properties of *PIEZO2*-positive cells. (J) CellChat analysis revealed a network in which *PIEZO2*-positive cells emit diverse outgoing signals to immune cells. This result indicated potential abnormalities in regulating or suppressing immune responses mediated by these cells. This circle plot shows the intercellular communication between immune cells and *PIEZO2*-positive cells that send signals to them in keloid groups. The width of each edge indicates the number of ligand–receptor pairs. *PIEZO2*-positive cells send outgoing signals to diverse immune cell types, including CD8⁺ T cells, macrophages, and cDCs. Among these, macrophages show the most significant signal reception. Moderate to mild outgoing signals are also directed toward memory-type CD4⁺ T cells, undifferentiated CD4⁺ T cells, pDCs, and B cells. These findings suggest that *PIEZO2*-positive cells may have diverse effects on the immune microenvironment within the stroma.


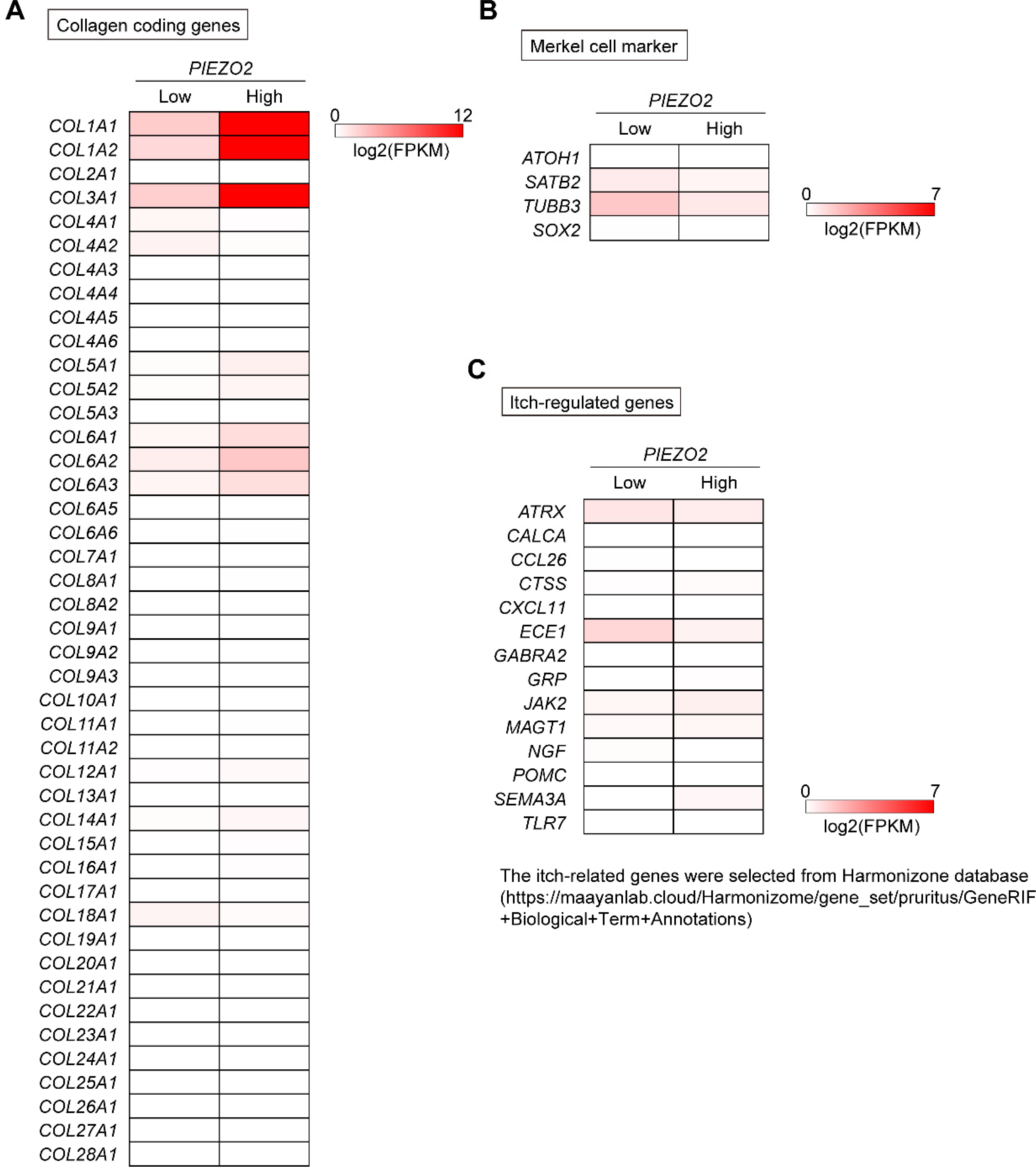


**Figure S4. Comparisons of gene expression trends between *PIEZO2*^hi^ and *PIEZO2*^lo^ cells.** (A–C) Supplementary results for the analysis presented in Figure 4. (A) *PIEZO2*^hi^ fibroblasts expressed various collagen genes more highly than *PIEZO2*^lo^ fibroblasts. (B) *PIEZO2*^hi^ fibroblasts showed low expression levels of other markers of Merkel cells. (C) *PIEZO2*^hi^ fibroblasts showed low expression levels of the chemical itch-related genes.


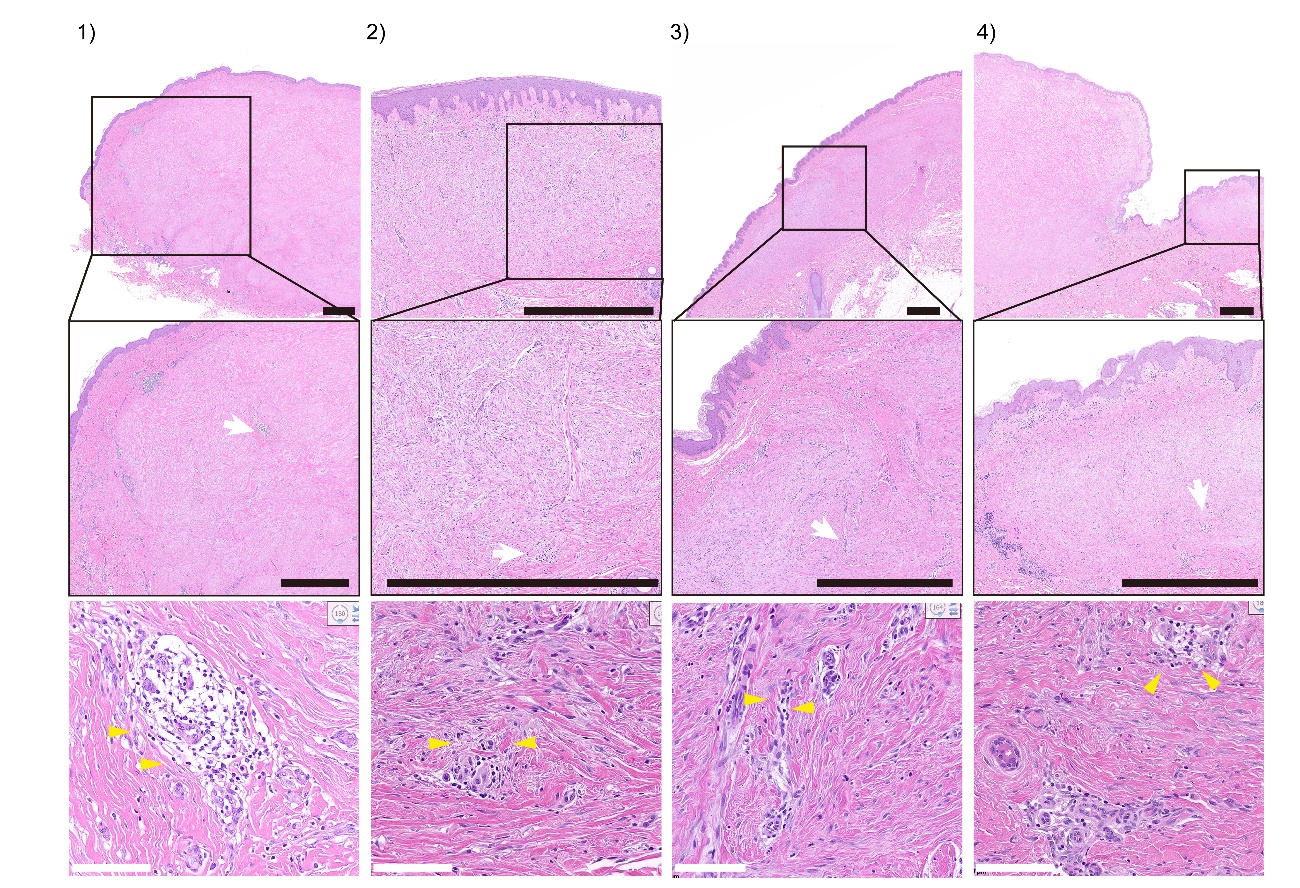


**Figure S5. Hematoxylin and eosin staining showing the histological appearance of specimens prepared from excised keloids.** The keloid patient case numbers are, from left to right, (1) case 14, (2) case 4, (3) case 25, and (4) case 11. Patient details are provided in supplementary material, Table S1. The upper and middle images demonstrate characteristic fibro-collagenous tissue and distribution in the dermis of each keloid case. The positions of the white arrows are identical to those demonstrated by the white arrows in Figure 5A. Scale bars (black lines, upper and middle panels): 1,000 µm. Yellow arrowheads in the lower-panel images point out areas where small cells appear around blood vessels or lymphatic vessels, and in the widened (or separated) space between haphazardly arranged thick collagen fibers in the dermis. Scale bars (white lines, lower panels): 100 µm.


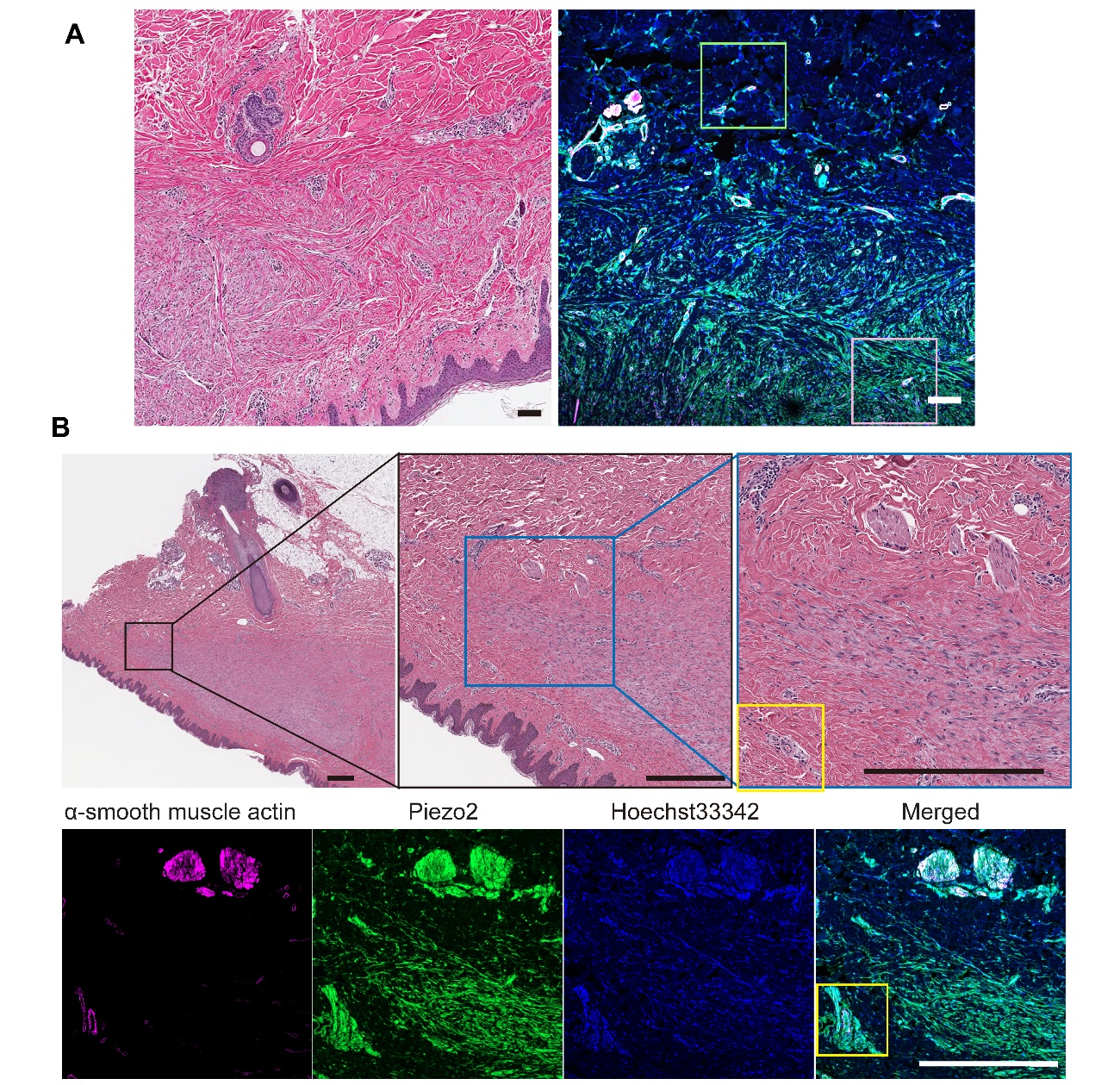


**Figure S6.** **Histological analysis using H&E staining and merged images from multiplex immunofluorescence staining showing PIEZO2-positive cells.** Supplementary results supporting the findings presented in Figure 5B. (A) H&E staining and the merged image with multiplex immunofluorescence staining (PIEZO2-positive cells: green; Hoechst 33342: blue; αSMA: cyan) on serial sections. Characteristic fibro-collagenous proliferation in the dermis and densely clustered inflammatory cells in the dermis are observed. The squares outlined in green and pink include the inactively and actively growing areas of keloid demonstrated in the upper and middle low images in Figure 5B, respectively. Scale bars (both black and white lines): 100 µm. (B) H&E staining and the merged image with the multiplex immunofluorescence staining (PIEZO2-positive cells: green; Hoechst 33342: blue; αSMA: cyan) on serial sections. Characteristic fibro-collagenous proliferation in the dermis and densely clustered inflammatory cells in the dermis are observed. The yellow rectangle indicates the actively growing areas of keloid demonstrated in the lower images of Figure 5B. Scale bars (both black and white lines): 500 µm.


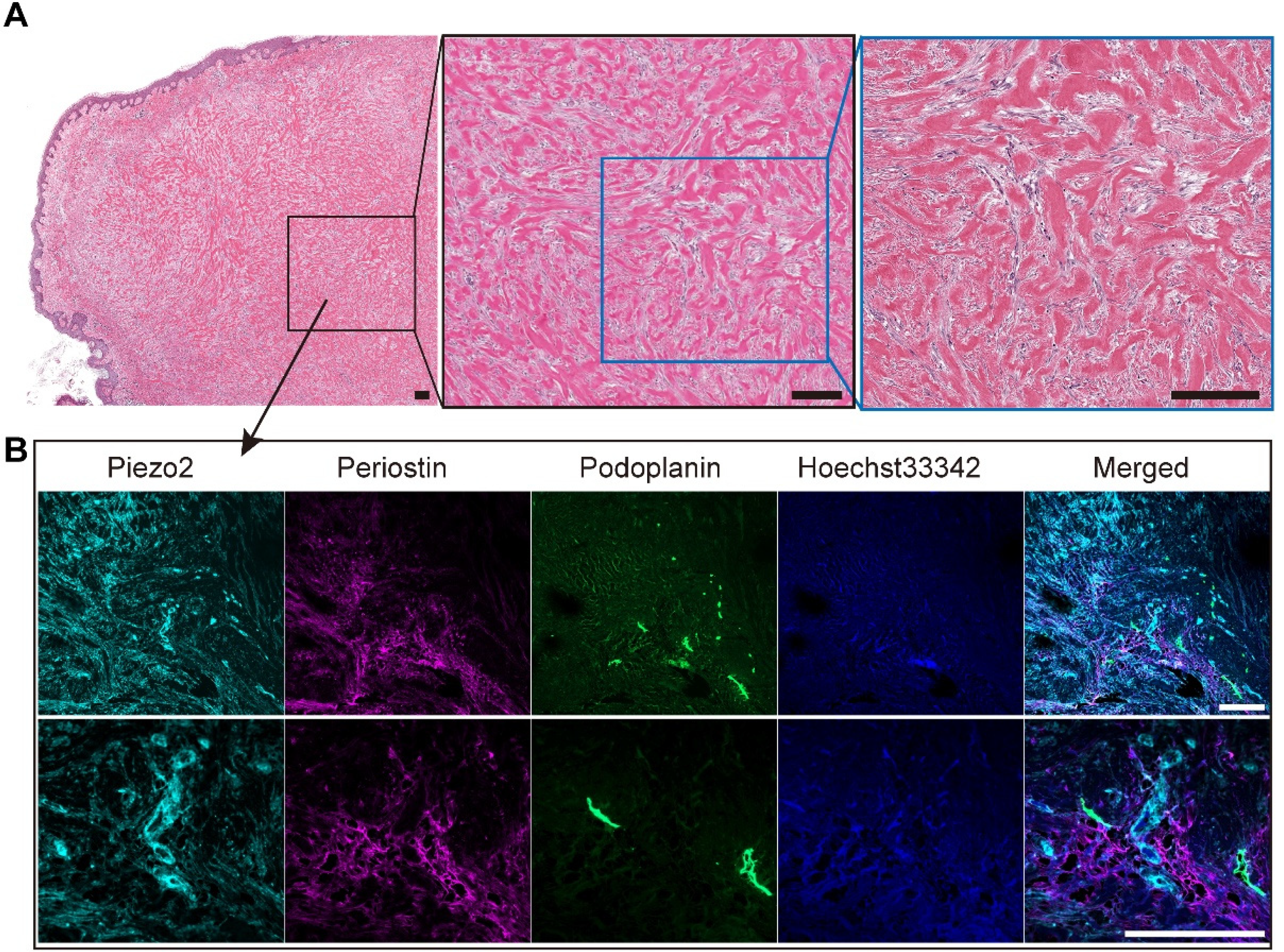


**Figure S7.** **Spatial distribution of PIEZO2, periostin, and podoplanin in keloid tissue.** Supplementary results supporting the findings in Figure 5C. (A) Hematoxylin and eosin (H&E) staining shows the histological architecture. (B) Multiplex immunofluorescence staining indicates the spatial distribution of Piezo2 (cyan), periostin (magenta), and podoplanin (green) positive cells corresponding to the square outlined in black indicated in panel A. Scale bars: 200 µm for both black and white lines.


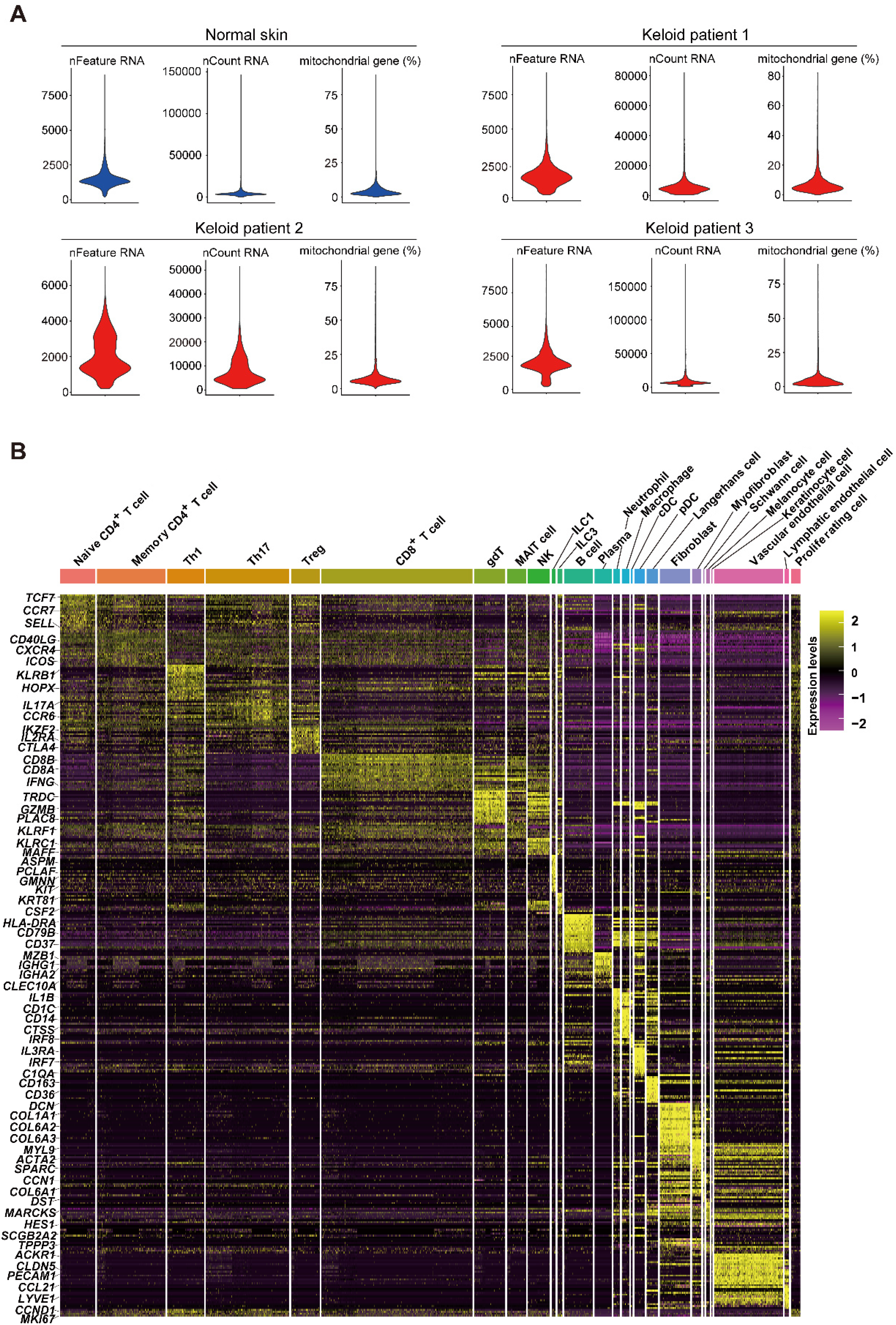


**Figure S8. Quality control and expression of marker genes for each cluster in single-cell RNA sequencing (scRNA-seq).** (A) Cells with nFeature RNA with < 1,000 genes, nFeature RNA with > 4,000 genes and > 10% mitochondrial gene expression were excluded. (B) Marker genes per cluster.

**Table S1.** Details of patients with keloids (cases KL1–KL10), severe lymphedema (SL1–SL10), and mild lymphedema (ML1–ML10) used for gene expression level comparison.

| **Case** | **Sex** | **Age**  **(years)** | **Duration of disease (months)** | **Location** | **JSS score** |
| --- | --- | --- | --- | --- | --- |
| KL1 | F | 25 | 60 | Ear lobe | 18 |
| KL2 | M | 13 | 18 | Thoracic | 17 |
| KL3 | M | 57 | 64 | Thoracic | 16 |
| KL4 | M | 19 | 29 | Thoracic | 11 |
| KL5 | F | 36 | 12 | Thoracic | 17 |
| KL6 | F | 39 | 24 | Abdomen | 11 |
| KL7 | M | 25 | 49 | Ear lobe | 10 |
| KL8 | F | 19 | 38 | Abdomen | 10 |
| KL9 | F | 68 | 25 | Abdomen | 9 |
| KL10 | M | 18 | 29 | Thoracic | 14 |
| **Case** | **Sex** | **Age (years)** | **ISL stage** | **Lymphoscintigraphy type** | |
| SL1 | M | 74 | II Late | IV | |
| SL2 | F | 63 | II | V | |
| SL3 | F | 63 | II | V | |
| SL4 | F | 62 | III | V | |
| SL5 | M | 58 | II Late | V | |
| SL6 | F | 53 | II Late | V | |
| SL7 | M | 17 | III | V | |
| SL8 | F | 67 | III | V | |
| SL9 | F | 74 | II | IV | |
| SL10 | F | 78 | III | V | |
| ML1 | F | 67 | Normal | Normal | |
| ML2 | M | 59 | Normal | Normal | |
| ML3 | F | 56 | I | II | |
| ML4 | F | 62 | I | II | |
| ML5 | F | 46 | I | II | |
| ML6 | F | 33 | 0 | I | |
| ML7 | F | 38 | 0 | I | |
| ML8 | F | 38 | 0 | I | |
| ML9 | F | 70 | I | II | |
| ML10 | F | 80 | I | II | |

JSS, Japan Scar Workshop (JSW) Scar Scale; ISL stage, lymphedema disease staging according to the International Society of Lymphology [63].

**Table S2.** Details of patients with keloids (cases 11–26).

| **Case No.** | **Sex** | **Age (years)** | **Duration of disease**  **(months)** | **Location** | **JSS score** | **Analysis** |
| --- | --- | --- | --- | --- | --- | --- |
| 11 | M | 55 | 228 | Thoracic | 18 | scRNA-seq (proliferative site) |
| 12 | F | 71 | 29 | Abdomen | 13 | scRNA-seq (proliferative site and normal skin) |
| 13 | M | 61 | 61 | Thoracic | 14 | scRNA-seq (proliferative site) |
| 14 | M | 11 | 20 | Mandibular | 19 | FACS |
| 15 | F | 24 | 36 | Ear lobe | 11 | Small cell detection |
| 16 | F | 73 | 111 | Thoracic | 14 | Small cell detection |
| 17 | F | 74 | 120 | Thoracic | 16 | Small cell detection |
| 18 | F | 29 | 96 | Ear lobe | 10 | Small cell detection |
| 19 | F | 74 | 112 | Thoracic | 17 | Small cell detection |
| 20 | F | 71 | 216 | Thoracic | 18 | Small cell detection |
| 21 | M | 49 | 22 | Thoracic | 16 | Small cell detection |
| 22 | F | 71 | 300 | Thoracic | 19 | Small cell detection |
| 23 | M | 23 | 36 | Thoracic | 17 | Small cell detection |
| 24 | F | 71 | 120 | Abdomen | 19 | TEM |
| 25 | F | 44 | 96 | Abdomen | 12 | TEM |
| 26 | M | 52 | 120 | Thoracic | 13 | TEM |

JSS, Japan Scar Workshop (JSW) Scar Scale [19]; TEM, transmission electron microscopy.

**Table S3.** Relative expression ratios of RNA (mean ± SE) in tissues from patients with mild lymphedema (ML), severe lymphedema (SL), and keloids.

| **Gene** | **ML**  **(*n* = 10)** | **SL**  **(*n* = 10)** | **Keloids**  **(*n* = 10)** |
| --- | --- | --- | --- |
| *PIEZO1* | 2,669 ± 545 | 1,918 ± 188 | 1,893 ± 193 |
| *PIEZO2* | 158 ± 35 | 223 ± 42 | 1,958 ± 633 |
| *POSTN* | 6,979 ± 1,071 | 5,456 ± 862 | 106,386 ± 24,177 |
| *COL1A2* | 84,820 ± 15,125 | 171,487 ± 60,541 | 1,560,280 ± 535,485 |

**Table S4.** Results of Kruskal–Wallis test comparing the mild lymphedema, severe lymphedema, and keloid groups.

| **Gene** | **Df** | **Sum of squares** | **Mean square** | ***F* value** | ***p* value** |
| --- | --- | --- | --- | --- | --- |
| *PIEZO1* | 2 | 3,891,217 | 1,945,608 | 1.5781 | 0.2248 |
| *PIEZO2* | 2 | 20,857,849 | 10,428,295 | 10,428,925 | <0.0001 |
| *POSTN* | 2 | 6.6903 × 10^10^ | 3.345 × 10^10^ | 17.1128 | <0.0001 |
| *COL1A2* | 2 | 1.3711 × 10^13^ | 6.855 × 10^12^ | 7.0762 | 0.0034 |

Df, degrees of freedom.

**Table S5.** Results of the Wilcoxon test comparing gene expression between the groups.

| **Gene** | **Difference of levels** | **Mean difference** | **SE** | **95% CI** | ***p* value** |
| --- | --- | --- | --- | --- | --- |
| *PIEZO2* | Keloid–ML  Keloid–SL  SL–ML | 1,800.555  1,735.250  65.305 | 358.4432  358.4432  358.4432 | 911.825–2,689.285  846.520–2,623.980  −823.425 to 954.035 | <0.0001  0.0001  0.9819 |
| *POSTN* | Keloid–ML  Keloid–SL  SL–ML | 100,930.0  99,406.7  1,523.2 | 19,772.50  19,772.50  19,772.50 | 51,905.7–149,954.2  50,382.4–148,431.0  −47,501.1 to 50,547.5 | <0.0001  <0.0001  0.9967 |
| *COL1A2* | Keloid–ML  Keloid–SL  SL–ML | 1,475,460  1,388,794  86,667 | 440,180.5  440,180.5  440,180.5 | 384,069–2,566,852  297,402–2,480,185  −1,004,725 to 1,178,058 | 0.0065  0.0106  0.9789 |

SE, standard error.

**Table S6.** Results of Pearson’s correlation coefficient test between two genes based on relative RNA expression ratios (*n* = 30).

| ***X*-axis** | ***Y*-axis** | ***R*** | **95% CI** | ***p* value** |
| --- | --- | --- | --- | --- |
| *PIEZO1* | *COL1A2* | −0.02622 | −0.38287 to 0.337234 | 0.8906 |
| *PIEZO1* | *POSTN* | −0.06153 | −0.41265 to 0.30551 | 0.7467 |
| *PIEZO2* | *COL1A2* | 0.9252 | 0.847381–0.964111 | <0.0001 |
| *PIEZO2* | *POSTN* | 0.9118 | 0.821291–0.957517 | <0.0001 |
